# Murinization of Internalin Extends Its Receptor Repertoire, Altering *Listeria monocytogenes* Cell Tropism and Host Responses

**DOI:** 10.1371/journal.ppat.1003381

**Published:** 2013-05-30

**Authors:** Yu-Huan Tsai, Olivier Disson, Hélène Bierne, Marc Lecuit

**Affiliations:** 1 Institut Pasteur, Biology of Infection Unit, Paris, France; 2 Inserm U1117, Paris, France; 3 Université Paris Diderot, Sorbonne Paris Cité, Paris, France; 4 Institut Pasteur, Unité des Interactions Bactéries Cellules, Paris, France; 5 Inserm, U604, Paris, France; 6 INRA, USC2020, Paris, France; 7 Institut Pasteur, French National Reference Center and World Health Organization Collaborating Center on Listeria, Paris, France; 8 Université Paris Descartes, Sorbonne Paris Cité, Institut Imagine, Paris, France; 9 Necker-Enfants Malades University Hospital, APHP, Division of Infectious Diseases and Tropical Medicine, Paris, France; University of Toronto, Canada

## Abstract

*Listeria monocytogenes* (*Lm*) is an invasive foodborne pathogen that leads to severe central nervous system and maternal-fetal infections. *Lm* ability to actively cross the intestinal barrier is one of its key pathogenic properties. *Lm* crosses the intestinal epithelium upon the interaction of its surface protein internalin (InlA) with its host receptor E-cadherin (Ecad). InlA-Ecad interaction is species-specific, does not occur in wild-type mice, but does in transgenic mice expressing human Ecad and knock-in mice expressing humanized mouse Ecad. To study listeriosis in wild-type mice, InlA has been “murinized” to interact with mouse Ecad. Here, we demonstrate that, unexpectedly, murinized InlA (InlA^m^) mediates not only Ecad-dependent internalization, but also N-cadherin-dependent internalization. Consequently, InlA^m^-expressing *Lm* targets not only goblet cells expressing luminally-accessible Ecad, as does *Lm* in humanized mice, but also targets villous M cells, which express luminally-accessible N-cadherin. This aberrant *Lm* portal of entry results in enhanced innate immune responses and intestinal barrier damage, both of which are not observed in wild-type *Lm*-infected humanized mice. Murinization of InlA therefore not only extends the host range of *Lm*, but also broadens its receptor repertoire, providing *Lm* with artifactual pathogenic properties. These results challenge the relevance of using InlA^m^-expressing *Lm* to study human listeriosis and *in vivo* host responses to this human pathogen.

## Author Summary

Co-evolution of microbes with their hosts can select stringently specific host-microbe interactions at the cell, tissue and species levels. *Listeria monocytogenes* (*Lm*) is a foodborne pathogen that causes a deadly systemic infection in humans. *Lm* crosses the intestinal epithelium upon the interaction of its surface protein InlA with E-cadherin (Ecad). InlA-Ecad interaction is species-specific, does not occur in wild-type mice, but does in transgenic mice expressing human Ecad and knock-in mice expressing humanized mouse Ecad. To study listeriosis in wild-type mice, InlA has been “murinized” to interact with mouse Ecad. Here, we demonstrate that in addition to interacting with mouse Ecad, InlA^m^ also uses N-cadherin as a receptor, whereas InlA does not. This artifactual InlA^m^-N-cadherin interaction promotes bacterial translocation across villous M cells, a cell type which is not targeted by InlA-expressing bacteria. This leads to intestinal inflammation and intestinal barrier damage, both of which are not seen in humans and humanized mouse models permissive to InlA-Ecad interaction. These results challenge the relevance of using InlA^m^-expressing *Lm* as a model to study human listeriosis and host responses to this pathogen. They also illustrate that caution must be exercised before using “murinized” pathogens to study human infectious diseases.

## Introduction

Co-evolution of microbes with their hosts can select stringently specific host-microbe interactions at the cell, tissue and species levels [1]. Species-specific host-microbe interactions, which are the rule rather than the exception, pose a challenge for the use of laboratory animal models to study human pathogens, including *Listeria monocytogenes* (*Lm*), the etiological agent of listeriosis, a deadly foodborne infection. *Lm* is able to actively cross the intestinal barrier, reach the systemic circulation and cross the blood-brain and placental barriers, leading to its dissemination to the central nervous system and the fetus [2].

The mouse is a genetically amenable model that is widely used to investigate human diseases [3], [4]. To obtain a mouse model in which the pathogenic properties of a given pathogen are similar to what is observed in human, species specificity can be circumvented by humanizing the mouse by transgenesis [5], [6], [7], [8], knock-in [9], knock-out [10] or xenograft techniques [11]. One can also adapt the pathogen to the mouse by multiple passages on cell lines [12], [13] or *in vivo* [14], or specifically “murinize” a pathogen ligand so that it interacts with the mouse ortholog of a species-specific human receptor [15], [16].

The *Lm* surface protein InlA interacts with E-cadherin (Ecad) and mediates *Lm* entry into epithelial cells, which express this adherens junction protein [17], [18]. Cadherins constitute a family of calcium-dependent cell adhesion receptors. Ecad is expressed mainly in epithelia, whereas N-cadherin (Ncad) is found primarily in neuronal cells and endothelial cells together with VE-cadherin [19], [20]. Ncad can also be coexpressed with Ecad in epithelial cells [21]. Importantly, Ncad has been reported to not act as a receptor for InlA, and so far Ecad is the only known classical cadherin acting as a receptor for InlA [18]. In contrast to Ecad from human, guinea pig, rabbit and gerbil, mouse Ecad (mEcad) and rat Ecad are not recognized by InlA and do not promote bacterial entry [9], [22]. The interaction of InlB, another *Lm* invasion protein, with its host receptor is also species-specific [23]. InlB recognizes the hepatocyte growth factor receptor Met of human, mouse, rat and gerbil but not that of guinea pig and rabbit [9], [23], [24].

Two mouse lines have been established to study InlA-Ecad interaction *in vivo*: a transgenic mouse line expressing human Ecad (hEcad) in enterocytes (hEcad Tg) [6], and a humanized mEcad knock-in mouse line (E16P KI) with an E16P amino acid substitution which enables mEcad to interact with InlA without affecting Ecad homophilic interactions and allows *Lm* internalization [9], [22]. Using these two humanized mouse models, we have demonstrated that InlA mediates *Lm* crossing of the intestinal epithelium upon targeting of luminally-accessible Ecad around goblet cells [6], [9], [25], and that InlA and InlB act interdependently to mediate the crossing of the placental barrier [9]. Epidemiological investigations have confirmed the relevance of these experimental findings, and shown that InlA is implicated in *Lm* crossing of human intestinal and placental barriers [9], [26].

In 2007, Wollert *et al.* engineered a genetically modified InlA with the purpose of increasing its binding affinity to hEcad [16]. Two amino acid substitutions in InlA, S192N and Y369S, were shown to enhance InlA binding affinity to hEcad [16]. Neither S192N nor Y369S substitution has been observed in the more than 500 *Lm* isolates InlA sequences we have checked (our unpublished results). Wollert *et al.* published that this increased affinity for hEcad translates into an increased bacterial entry into human epithelial cells (Caco-2) [16]. Importantly, Wollert *et al.* also showed that this modified InlA binds the extracellular cadherin domain 1 (EC1) of mEcad in solution with a comparable affinity to that of the wild-type (wt) InlA for hEcad EC1 [16]. They hypothesized that this interaction would allow *Lm* expressing this “murinized” InlA (InlA^m^) to cross intestinal barrier and would render wt mice orally permissive to *Lm* infection, a phenotype which is mediated by InlA in permissive models [6]. In support of this hypothesis, Wollert *et al.* found an increased intestinal, spleen and liver bacterial loads of wt mice orally inoculated with *Lm* expressing InlA^m^, yet only after 3 to 4 days post infection, which is later than in models permissive to InlA-Ecad interaction [6], [9], [16]. Moreover, the ability of InlA^m^ to mediate mEcad-dependent *Lm* internalization into host cells has never been tested. In addition, InlA^m^ unexpectedly promoted pronounced inflammation and intestinal epithelial cell damages in wt mice [16], whereas wt InlA mediates the crossing of the intestinal barrier without inducing significant intestinal response and tissue damage in hEcad transgenic mice [6], [27].

This prompted us to investigate the detailed properties of InlA^m^ in cultured cells, as well as the *in vivo* cell and tissue tropisms of bacteria expressing InlA^m^, as compared to that of its isogenic parental *Lm* strain that expresses wt InlA. Here, we demonstrate that InlA^m^ promotes bacterial entry not only into mEcad-positive but also into mEcad-negative mouse cells. We show that InlA^m^-mediated entry into mEcad-negative cells is mouse Ncad (mNcad)-dependent. Importantly, InlA^m^-mNcad interaction allows bacteria to specifically target Ncad-positive villous M cells *in vivo*, a cell type which is not targeted by *Lm* in humanized mouse models permissive to InlA-Ecad interaction. This leads to enhanced intestinal inflammatory responses and disruption of the intestinal barrier integrity, both of which are not observed in *Lm*-infected humanized mice and human listeriosis. Together, these results demonstrate that the murinization of InlA not only extends *Lm* host range, but also broadens its receptor repertoire, consequently changing *Lm* cell tropism and enhancing host immune responses to *Lm*. These results challenge the relevance of using InlA^m^-expressing *Lm* to study human listeriosis and *in vivo* host responses to this human pathogen.

## Results

### Murinization of InlA promotes bacterial entry into mEcad-expressing cells but has no impact on bacterial entry into hEcad-expressing cells

**Figure 1:**
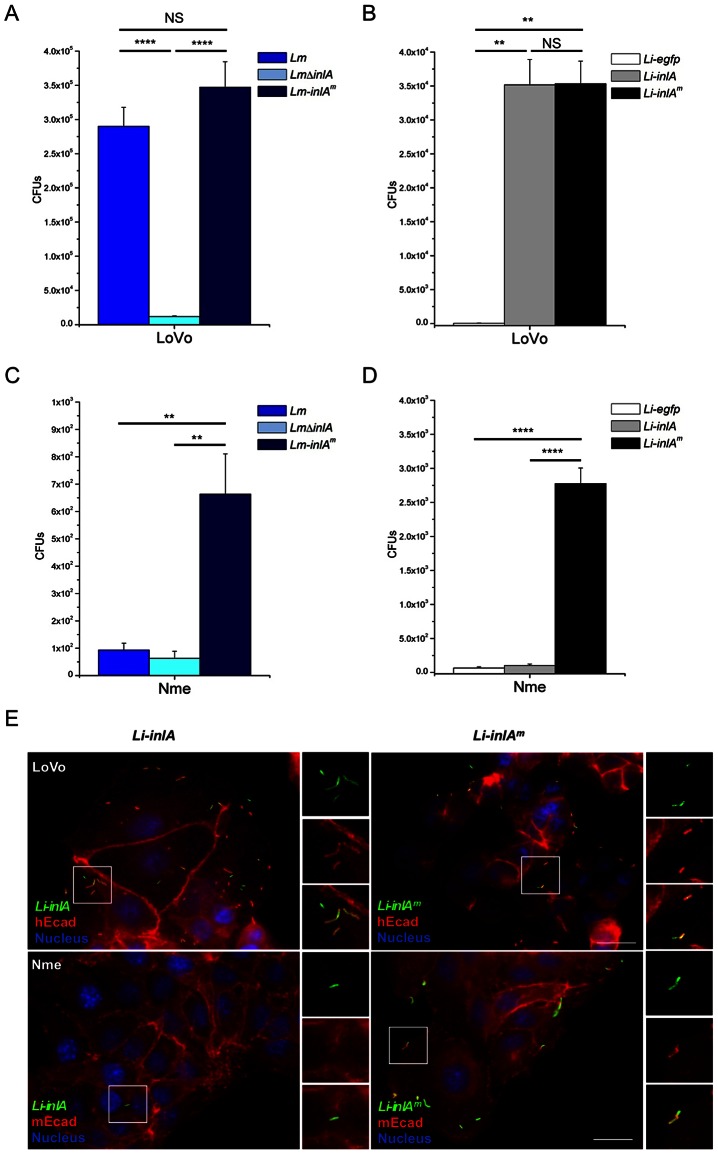
Respective ability of InlA and InlA^m^ to promote bacterial entry into hEcad- and mEcad-expressing cells. Bacterial entry into hEcad-expressing human epithelial cells (LoVo) (A for *Lm* and B for *Li*) and mEcad-expressing mouse epithelial cells (Nme) (C for *Lm* and D for *Li*) was performed by counting intracellular gentamicin resistant bacteria following 1 hr of infection and 1 hr of gentamicin incubation. No significant difference was observed between InlA- and InlA^m^-expressing bacteria when invading LoVo cells, whereas InlA^m^ expression promoted bacterial entry into Nme cells. Values are expressed as a mean + SD (n = 3). Statistical analysis was performed with the unpaired Student's *t* test. (E) Recruitment of Ecad was performed by incubating *Li-inlA* or *Li-inlA^m^* with the cells cultured on coverslips for 1 hr, followed by PBS wash and fixation before staining. The cells were stained with the anti-*Li* antibody and anti-hEcad or anti-mEcad antibody. Right panels show separated channels and merge of boxed regions, showing the recruitment of cadherin by bacteria. Both *Li-inlA* and *Li-inlA^m^* recruit hEcad when incubated with LoVo cells. *Li-inlA^m^* but not *Li-inlA* is able to recruit endogenous mEcad in Nme cells. Scale bar, 20 µm.

We first investigated whether the increased affinity of InlA^m^ to hEcad translates into an enhanced invasion of hEcad-expressing cells, as proposed by Wollert *et al.* [16]. To this end, we assessed InlA^m^-dependent entry into LoVo cell, a human epithelial cell line expressing hEcad [22]. *Lm* wt strain and *Lm* expressing InlA^m^ (*Lm*-*inlA^m^*) invaded LoVo cells at similar levels (Figure 1A). Because *Lm* can be internalized by InlA-independent pathways such as InlB-Met, we transferred either *inlA* or *inlA^m^* onto the chromosome of *Listeria innocua* (*Li*), a naturally non-invasive and non-pathogenic *Listeria* species, in which heterologous expression of *inlA* has been shown to confer invasiveness [17], [18], [28]. *Li* expressing either InlA (*Li*-*inlA*) or InlA^m^ (*Li*-*inlA^m^*) were equally invasive in LoVo cells (Figure 1B). These results indicate that contrary to what is reported by Wollert *et al.* [16], the increased affinity of InlA^m^ to hEcad does not translate into an increased level of bacterial entry. Both *Li-inlA* and *Li-inlA^m^* recruited hEcad when incubated with LoVo cells, suggesting that hEcad is involved in both InlA- and InlA^m^-mediated entries (Figure 1E, upper panel). Because purifed InlA^m^ interacts with the purified EC1 domain of mEcad, Wollert *et al.* have proposed, although not tested, that InlA^m^ would mediate bacterial entry into mEcad-expressing cells [16]. We therefore tested the ability of InlA^m^ to promote bacterial entry into the mouse epithelial cell line Nme, which expresses mEcad [29]. InlA^m^ promoted bacterial entry into mEcad-expressing Nme cells, although to a lower level than InlA in hEcad-expressing LoVo cells (Figure 1C and D). *Li*-*inlA^m^* also recruited mEcad during cell invasion, whereas as expected, *Li*-*inlA* does not (Figure 1E, lower panel). Together, these results show that (*i*) the increased affinity of InlA^m^ to hEcad does not enhance bacterial entry into hEcad-expressing cells, and (*ii*) the murinization of InlA confers to *Lm* an enhanced ability to be internalized into mEcad-expressing cells [16].

### InlA^m^ promotes mEcad-independent entry into mouse cells

**Figure 2:**
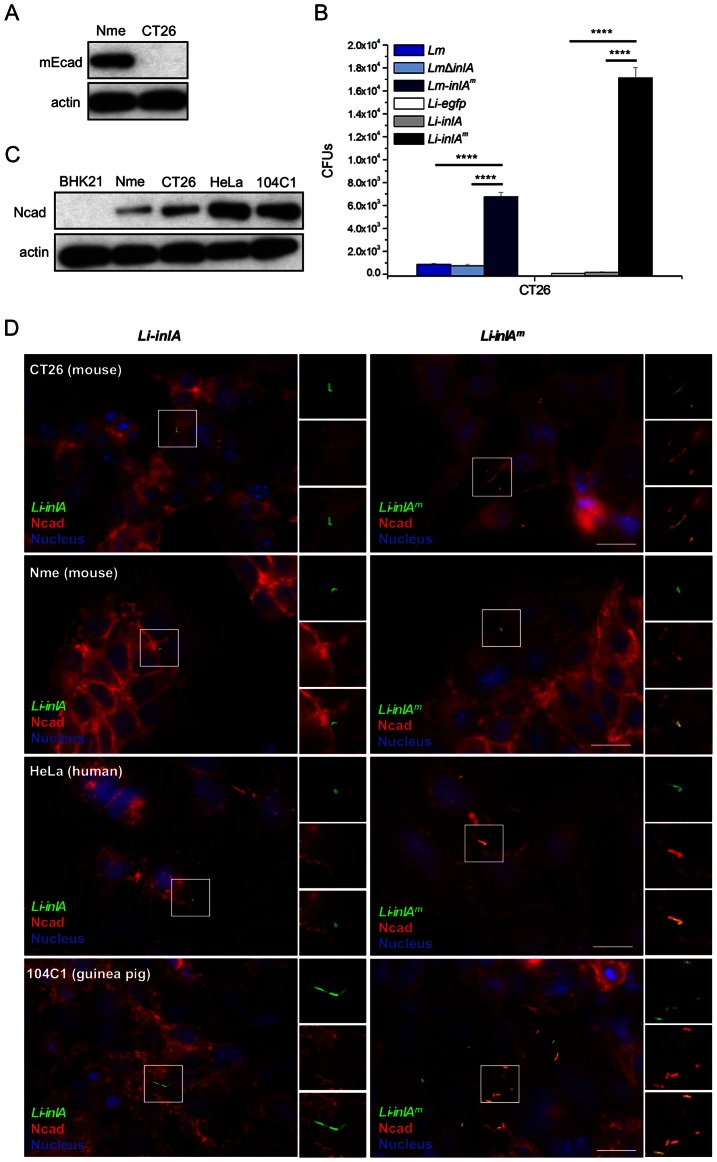
InlA^m^ promotes bacterial entry into mEcad-negative mouse cells and recruits endogenous Ncad. (A) Nme cells express mEcad, whereas no mEcad expression can be detected in CT26 cells. Actin expression was used as a loading control. (B) Bacterial entry was performed as described in Figure 1. InlA^m^- but not InlA-expression promotes bacterial entry into mouse CT26 cells. Values are expressed as a mean + SD (n = 3). Statistical analysis was performed with the unpaired Student's *t* test. (C) Nme cells, CT26 cells, HeLa cells and 104C1 cells express Ncad. The lysate of BHK21 cells which do not express any detectable classical cadherins was used as a negative control. (D) Recruitment of Ncad was performed as described in Figure 1. The coverslips were stained with the anti-*Li* antibody and anti-Ncad antibody. Right panels show separated channels and merge of boxed regions, showing the recruitment of Ncad by *Li-inlA^m^*. Scale bar, 20 µm. See also Figures S1 and S2.

Monk *et al*. have reported that *Lm*-*inlA^m^* invades mouse CT26 cells more efficiently than *Lm* [13]. Strikingly, CT26 cells do not express mEcad (Figure 2A) [30], yet we confirmed that InlA^m^ mediates bacterial entry into these cells (Figure 2B). Because classical cadherins exhibit a high level of conservation in their EC1 domains (Figure S1A), we tested whether *Li-inlA^m^* would recruit another classical cadherin than mEcad in CT26 cells. We labeled CT26 cells with a pan-cadherin antibody, which recognizes the cytoplasmic domain of classical cadherins [31]. CT26 cells were strongly stained with the pan-cadherin antibody (Figure S1B), indicating that they likely express classical cadherin proteins. Furthermore, this pan-cadherin-immunoreactive protein was recruited in CT26 cells by *Li-inlA^m^* but not *Li-inlA* (Figure S1B). Immunoblotting and immunostaining revealed that CT26 cells express Ncad (Figures 2C and D), a classical cadherin known to be expressed in endothelial cells, neurons and some transformed epithelial cells [20]. Importantly, *Li*-*inlA^m^*, but not *Li*-*inlA*, recruited Ncad in CT26 cells (Figure 2D). We next tested other cell lines for Ncad expression. We found that Nme cells (which also express mEcad and are permissive to InlA^m^-mediated entry), human HeLa cells, and guinea pig 104C1 cells all express Ncad (Figure 2C). As in CT26 cells, InlA^m^ promoted bacterial entry into HeLa and 104C1 cells, although these two cell lines do not express Ecad and are therefore not permissive to InlA-dependent entry (Figure S2) [23]. These results suggest that the murinization of InlA confers to this protein the ability to interact with Ncad from different species, and to enter into host cells expressing Ncad.

### mNcad is a receptor for InlA^m^ but not InlA

**Figure 3:**
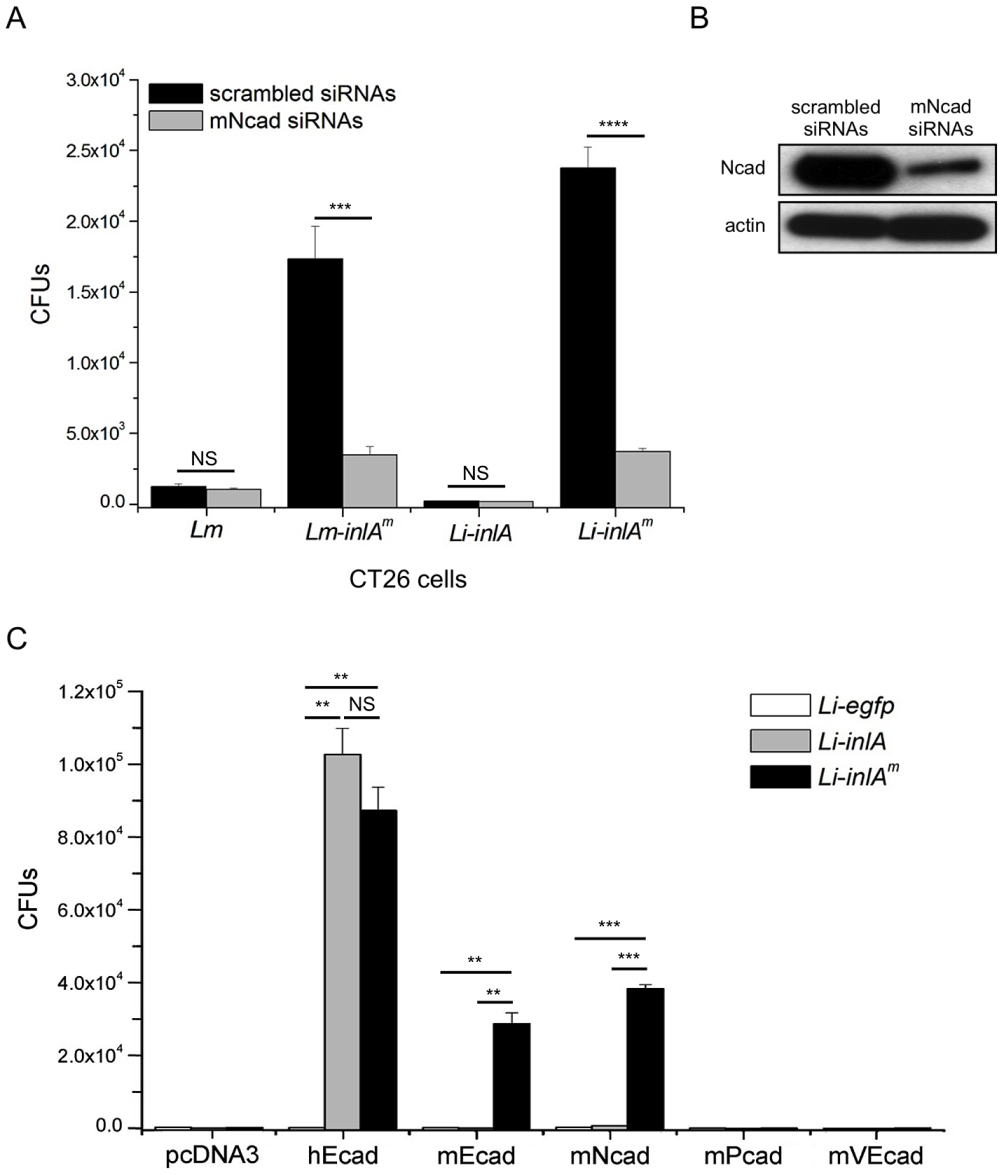
InlA^m^ mediates mouse Ncad-dependent internalization. (A) Mouse CT26 cells were transfected with scrambled siRNAs or mNcad-specific siRNAs. Bacteria internalization was evaluated by counting intracellular gentamicin resistant bacteria. Values are expressed as a mean + SD (n = 3). Statistical analysis was performed with the unpaired Student's *t* test. (B) The expression of mNcad was decreased in the cells transfected with mNcad–specific siRNAs compared to that transfected with scrambled siRNAs. Decrease of mNcad expression in CT26 cells reduced the entry of InlA^m^-expressing bacteria but not that of InlA-expressing bacteria into CT26 cells. (C) To evaluate the function of different cadherins as receptors, BHK21 cells were transiently transfected with pcDNA3 expression vector harboring the cDNAs of each cadherin. Bacterial invasion were evaluated by counting intracellular gentamicin resistant bacteria. Expression of hEcad provides gain-of-function for both InlA- and InlA^m^-expressing *Li* to invade. InlA^m^ but not InlA expression promotes bacterial entry to mEcad- and mNcad-expressing cells. Neither mouse P-cadherin (mPcad) nor mouse VE-cadherin (mVEcad) expression promotes InlA- and InlA^m^-dependent entry. Values are expressed as a mean + SD (n = 3). Statistical analysis was performed with the unpaired Student's *t* test. See also Figure S1.

To investigate if mNcad serves as a receptor for InlA^m^-mediated entry into CT26 cells, CT26 cells were treated with mNcad-specific siRNAs or scrambled control siRNAs. Treatment of CT26 cells with mNcad siRNAs led to a reduced expression of mNcad which correlated with a significantly decreased InlA^m^-dependent entry (Figures 3A and B). To directly assess the ability of mEcad and mNcad to act as receptors for InlA^m^, we used the BHK21 cell line, which is of hamster origin and does not express any known classical cadherin [32], and transfected this cell line with plasmids encoding either hEcad, mEcad or mNcad. As expected, both InlA and InlA^m^ mediated bacterial entry into hEcad-expressing cells (Figure 3C). Moreover, InlA^m^ mediated entry into mEcad-expressing cells, whereas as previously shown, InlA did not (Figure 3C) [22]. Most importantly, we also demonstrated that InlA^m^ mediated bacterial entry into Ncad-expressing cells, whereas, as previously shown, InlA did not (Figure 3C) [18].

To investigate whether the InlA^m^ receptor repertoire extends to other members of classical cadherins, we tested the ability of mouse P-cadherin (mPcad) and VE-cadherin (mVEcad) to serve as receptors for InlA^m^ (Figure S1A). Neither mPcad nor mVEcad acted as a receptor for InlA^m^ or InlA (Figure 3C). Taken together, these data confirm that InlA exhibits a species-specific and narrow repertoire for Ecad and mediates entry into hEcad- but not mEcad-expressing cells, and demonstrate that by widening InlA species spectrum from human to mouse Ecad, murinization of InlA extends its receptor repertoire to Ncad.

### Murinization of InlA extends the cell tropism of *Lm* at the intestinal level

**Figure 4:**
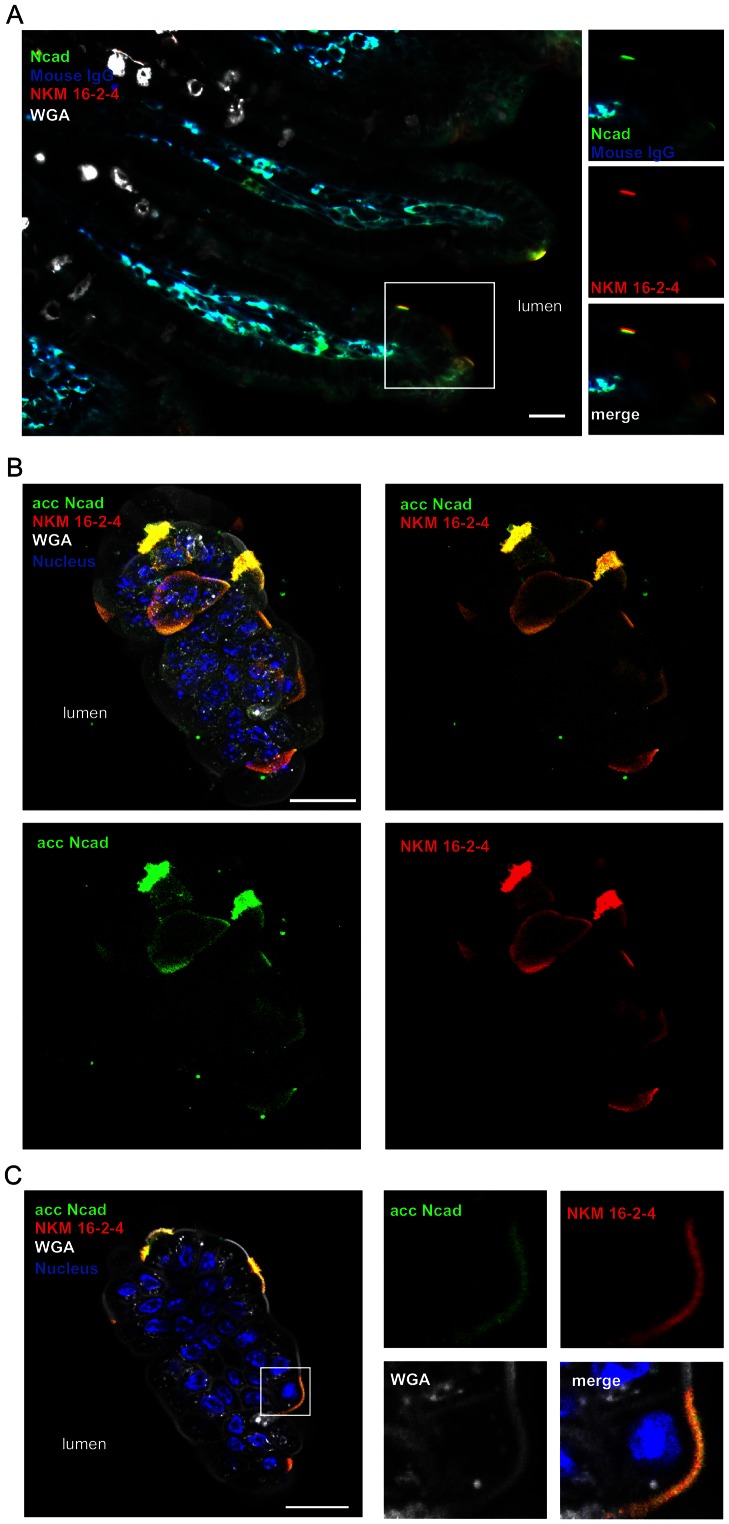
Ncad expression is detected on the apical pole of villous M cells. Immunolabeling of the small intestine tissue section (A) and whole mount tissue of a part of ileum (B and C) of wt mice was performed. (A) The small intestine tissue section was stained by anti-mouse IgG followed by anti-Ncad mouse IgG and anti-mouse IgG conjugated with another fluorophore to distinguish mouse IgG-positive cells and Ncad-positive cells. Scale bar, 50 µm. (B and C) Intestinal tissue of wt mice was fixed and stained for luminally accessible (acc) Ncad with antibody against extracellular domain of Ncad (clone GC4) before tissue permeabilization, M cells with NKM 16-2-4 antibody, wheat germ agglutinin (WGA) and nuclei after tissue permeabilization. Projection of a 25 µm thick reconstructed intestinal villus (B) and one of the xy plane (C) are shown. Right panels show separated channels and merge of boxed regions in (C), showing Ncad on the apical side of NKM 16-2-4-positive cells. NKM 16-2-4 antibody is a monoclonal antibody raised against α(1,2) fucose moiety in absence of neighboring sialic acids, a specific marker on M cells surface. WGA was used to stain the mucus of goblet cells and cell membrane. Scale bar, 20 µm. See also Figure S3, Movies S1, S2 and S3.

In order to investigate if these *in vitro* results translate into an *in vivo* phenotype, and study in particular the cell tropism of InlA^m^-expressing bacteria, we investigated Ncad luminal accessibility at the intestinal epithelium level, which is the portal of InlA-mediated entry of *Lm*. In contrast to luminally-accessible Ecad which is mostly observed as rings surrounding goblet cells [25], mNcad was accessible on the apical pole of villous M cells (Figure 4, Movie S1), but not M cells of Peyer's patches (Movie S2) in wt mice. The expression of luminally-accessible Ncad was also detected on the apical pole of villous M cells in E16P KI mice (Figure S3, Movie S3). These results suggest that InlA^m^ may allow bacteria to target villous M cells upon mouse oral inoculation.

**Figure 5:**
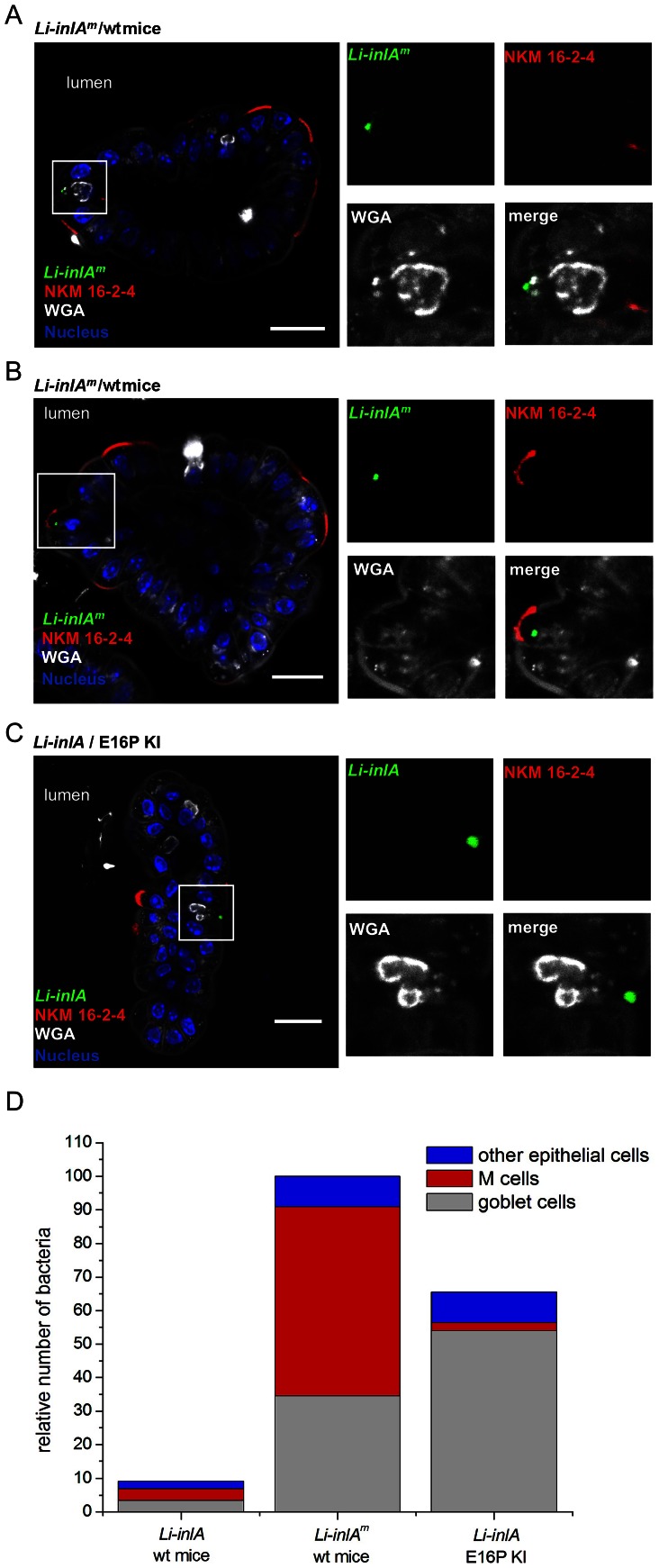
InlA^m^ mediates targeting of villous M cells. The intestinal ileum was taken from E16P KI mice and wt mice orally inoculated by 10^10^ *Li-inlA* and *Li-inlA^m^* at 5 hr post infection, respectively. The intestinal tissues were fixed and stained with WGA for goblet cells, NKM 16-2-4 monoclonal antibody for M cells, and for bacteria and nuclei. (A and B) The confocal Z-plane of an ileal villus from *Li-inlA^m^* infected wt mice demonstrates that *Li-inlA^m^* was able to target goblet cells (A) and villous M cells (B). Right panels show separated channels and merge of boxed regions, showing the location of bacteria in villous epithelia. (C) The confocal Z-plane of an ileal villus from *Li-inlA* infected E16P KI mice shows that *Li-inlA* targeted goblet cells. (D) Relative location of bacteria in mice intestinal epithelia of villi is shown. The total number of *Li-inlA^m^* in wt mice intestinal villi epithelia was set to 100. 20 villi from two mice ileal loops were counted in each set. Scale bar, 20 µm. See also  Figures S4, S5, S6, Movies S4, S5, S6, S7.

To specifically investigate whether InlA^m^-expressing bacteria target cells that express luminally-accessible Ncad, we inoculated orally wt mice with *Li-inlA* or *Li-inlA^m^*, and for comparison we inoculated humanized E16P KI mice orally with *Li-inlA*. As expected from our recent results [25], *Li-inlA* were found in goblet cells 5 hrs post oral inoculation of E16P KI mice (Figures 5C and D). In contrast, *Li-inlA^m^* targeted both goblet cells (Figures 5A and D) and villous M cells (Figures 5B and D, Movie S4) in wt mice. We next performed a detailed quantification of the location of bacteria in the intestinal epithelium (*i.e.* goblet cells, villous M cells, other epithelial cells). This demonstrated that, contrary to InlA, which targets almost exclusively goblet cells in E16P KI mice (82%), InlA^m^ preferentially targets villous M cells (56%) in wt mice, and to a lower degree goblet cells (34%) (p<0.001, χ^2^ test analysis) (Figure 5D). In agreement with these results obtained with *Li-inlA^m^*, *Lm-inlA^m^* also targeted both goblet cells (Figures S4A and D, S5A, Movie S5) and villous M cells (Figures S4B and D, S5B, Movie S6) in both wt and E16P KI mice, in contrast to *Lm* which exclusively targeted goblet cells, only in E16P KI mice (Figures S4C and D, S5C, Movie S7). Together, these results demonstrate that while InlA- and InlA^m^-Ecad interactions both contribute to the targeting of goblet cells, InlA^m^-mNcad interaction allows bacteria to target villous M cells, a cell type which is not targeted when InlA interacts only with its native receptor Ecad.

### InlA^m^-mNcad interaction has an impact on *Lm* systemic dissemination in orally inoculated mice

**Figure 6:**
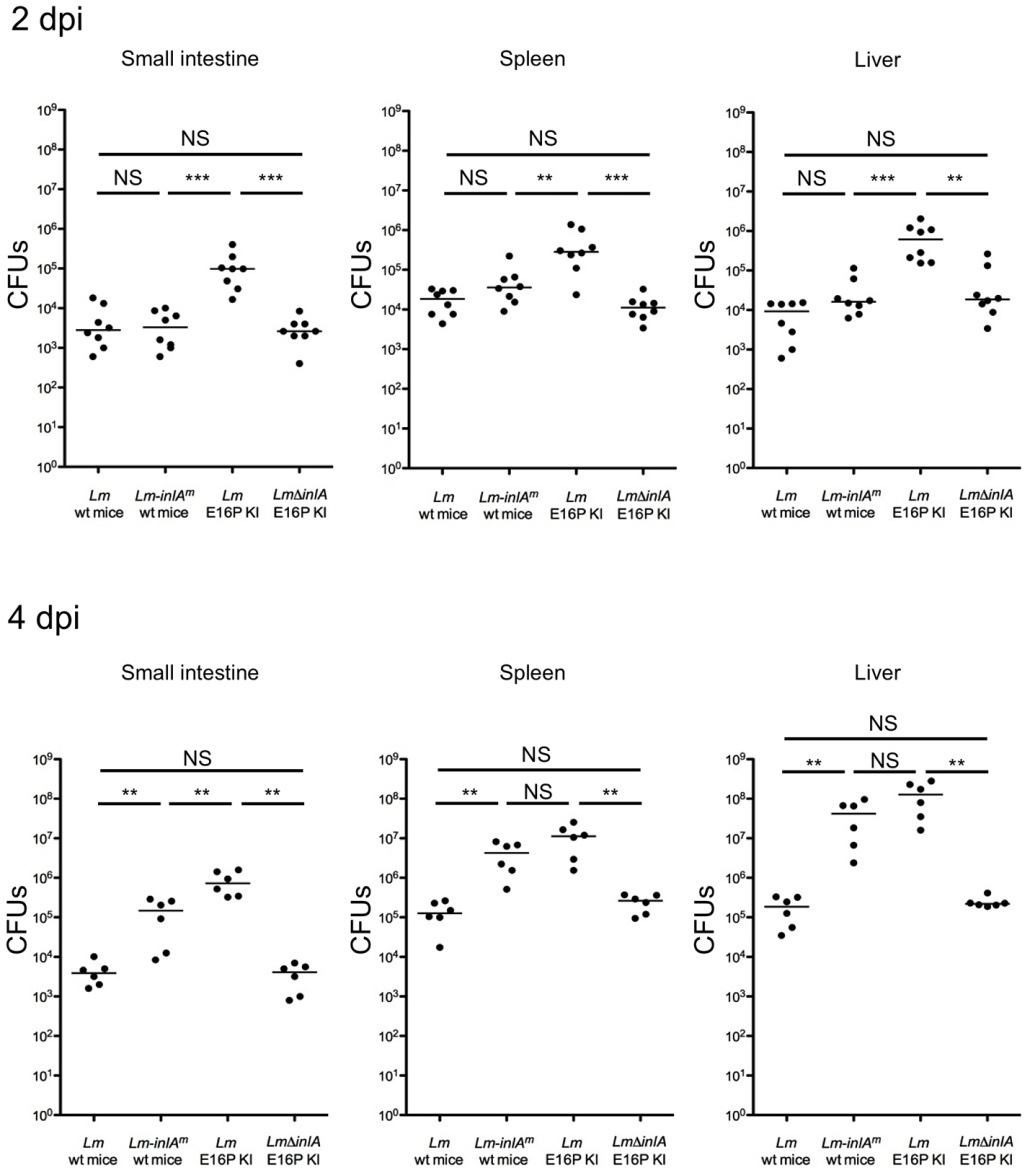
Respective invasive potential of *Lm* and *Lm*-*inlA* ^m^ in orally inoculated E16P KI and wt mice. Mice were orally inoculated with 10^10^ bacteria for 2 (n = 8, upper panel) or 4 (n = 6, lower panel) days. Bacterial loads in the ileum loops of small intestine, in spleens and in livers were shown. Statistical analysis was performed with the Mann-Whitney *u* test. See also Figure S7.

To investigate the impact of InlA^m^-mNcad interaction on the infection process, we inoculated orally wt and E16P KI mice with *Lm*-*inlA^m^* or *Lm*. In *Lm*-infected E16P KI mice in which InlA-Ecad interaction is functional, InlA promoted *Lm* invasion of the small intestinal tissue and bacterial dissemination to spleen and liver as early as 2 days post infection (dpi) (Figure 6). In contrast, in *Lm*-*inlA^m^* infected wt mice, in which both InlA^m^-Ecad and InlA^m^-Ncad interactions are functional, *Lm* bacterial loads in the small intestinal tissue, spleen and liver were not significantly increased at 2 dpi compared to *Lm*-infected wt mice, but were at 4 dpi (Figure 6). This delayed systemic dissemination was also observed when comparing *Lm*-*inlA^m^* to *Lm*Δ*inlA* in E16P KI mice (Figure S7). These results demonstrate that, although promoting *Lm* crossing of the wt mouse intestinal barrier, InlA^m^ delays bacterial systemic dissemination relative to InlA in E16P KI mice, and therefore alters the kinetics of *Lm* infection *in vivo*.

### InlA^m^-mNcad interaction leads to enhanced intestinal response and compromised intestinal barrier function

**Figure 7:**
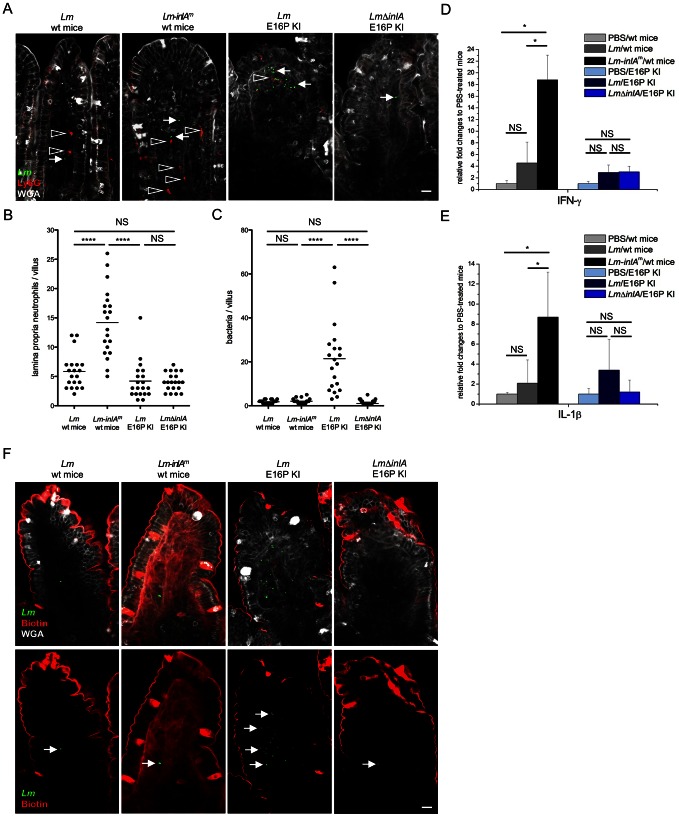
*Lm* expressing InlA^m^ stimulate inflammatory response in the small intestine and compromise intestinal epithelial barrier integrity. The intestinal ileum was taken from E16P KI and wt mice orally inoculated by 10^10^ *Lm* and *Lm-inlA^m^* at 24 (A–C) or 48 (D–F) hr post infection, respectively. (A) Anti-Ly6G antibody staining indicates neutrophils (red, highlighted by the open arrowheads). Tissues were stained for *Lm* (green, highlighted by the arrows) and counterstained with WGA (grey) for goblet cells, respectively. Scale bar, 20 µm. (B) No obvious difference on neutrophil numbers was observed between wt and E16P KI mice infected by *Lm*, whereas orally *Lm-inlA^m^* infection in wt mice induced neutrophil recruitment at 24 hr post infection. Statistical analysis was performed with the Mann-Whitney *u* test (n = 20 from 2 mice). (C) No obvious difference on bacterial numbers was observed between *Lm* and *Lm-inlA^m^* in wt mice intestinal villi, whereas a significantly increased invasion of *Lm* in the villi was obsevred in E16P KI mice at 24 hr post infection. Statistical analysis was performed with the Mann-Whitney *u* test (n = 20 from 2 mice). (D and E) RNA was extracted from the ileum loops of infected or PBS-treated mice 48 hr post infection (n = 4). Following reverse transcription reaction, gene expression was quantified by qPCR with normalization to the GAPDH transcript. Values are expressed as a mean + SD of the fold change relative to that in PBS-treated mice. No significant difference on IFN-γ (D) and IL-1β (E) expression was observed among PBS-treated, *Lm* and *Lm*Δ*inlA*-infected E16P KI mice. In contrast, *Lm*-*inlA^m^* oral infection induced 5 to 15 fold increase of IFN-γ and IL-1β gene expression in intestinal tissue compared to *Lm*-infected and PBS-treated wt mice. Statistical analysis was performed with the unpaired Student's *t* test. (F) Biotin (red) penetration into intestinal lamina propria was done to address intestinal barrier integrity during infection. Mice were sacrificed 2 days post infection. Biotin was injected into ileum loop followed by PBS wash and fixation. Tissues were stained for *Lm* (green, highlighted by the arrows) and counterstained with WGA (grey) for goblet cells, respectively. Biotin is located within lamina propria of the villi from *Lm*-*inlA^m^* infected mice but not *Lm* infected wt and E16P KI mice. Scale bar, 20 µm. See also Figure S8.

Given the changes in infection kinetics induced by InlA^m^, and the artifactual route of translocation taken by InlA^m^-expressing bacteria at the intestinal epithelium level, we investigated whether InlA^m^-Ncad-mediated targeting of villous M cells would have an impact on host responses. Strikingly, oral inoculation of *Lm*-*inlA^m^* led to a significant neutrophil recruitment in wt (Figures 7A and B), E16P KI (Figures S8A and B) and hEcad Tg mice (Figures S8A and B), which was not observed with *Lm* in E16P KI (Figures 7A and B) and in hEcad Tg mice (Figures S8A and B). Importantly, neutrophil infiltration correlated only with InlA^m^-mediated invasion, and did not reflect bacterial load in the villi, which was actually the highest in *Lm*-infected humanized mice, in which no neutrophil infiltration was observed (Figures 7A–C, S8A–C). Moreover, a significant increase in IFN-γ and IL-1β expression was observed in the intestinal tissue of wt mice infected with *Lm*-*inlA^m^*, whereas no significant increase was observed in *Lm*-infected wt and humanized mice (Figures 7 D and E). Together, these results indicate that InlA^m^-Ncad-mediated intestinal invasion *per se* leads to exacerbated host responses compared to InlA-Ecad-mediated intestinal invasion, and are not a reflect of enhanced bacterial tissue invasion.

We next assessed intestinal barrier integrity upon infection by testing the intratissular diffusion of biotin administered intraluminally (see Material and Methods) [33]. In wt and humanized mice infected by *Lm* for two days, biotin localized exclusively to the luminal side of the small intestine (Figures 7F and S8D). In contrast, although the intestinal villi of *Lm*-*inlA^m^* infected wt and humanized mice were not heavily infected, biotin accessed the lamina propria (Figures 7F and S8D). These findings indicate that InlA^m^-Ncad-mediated intestinal invasion leads to a disruption of intestinal barrier integrity. Together, these results demonstrate that the murinization of InlA profoundly modifies the pathogenic properties of *Lm* by altering its intestinal portal of entry, host intestinal responses and intestinal barrier integrity.

## Discussion

InlA interaction with Ecad allows *Lm* translocation across the intestinal epithelium and is therefore a critical event in the development of systemic listeriosis, one of the deadliest foodborne infections in human. Because InlA does not interact with mEcad, the discovery and characterization of this key step were made in species permissive to InlA-Ecad interaction (guinea pig, gerbil) and humanized mouse models (hEcad Tg and E16P KI mouse lines) [6], [9]. A genetically engineered *Lm* strain expressing a murinized InlA (InlA^m^) enabling interaction with mEcad *in vitro* has been proposed to constitute an attractive alternative model to study human listeriosis in wt mice [16]. A practical advantage of this latter system is that it can be readily used to infect several different mouse lines. However, a systematic study comparing the properties of *Lm* expressing InlA^m^ to that of its isogenic parental strain has not been performed, neither *in vitro* nor *in vivo*.

Here we show that InlA^m^ is able to recruit mEcad and mediate mEcad-dependent entry into cultured cells. We also show that InlA^m^ mediates entry into goblet cells of wt mice, which express luminally-accessible mEcad. These results confirm that the S192N and Y369S substitutions confer to InlA a phenotype in wt mice which is observed in humanized mice permissive to InlA-Ecad interaction [25].

**Figure 8:**
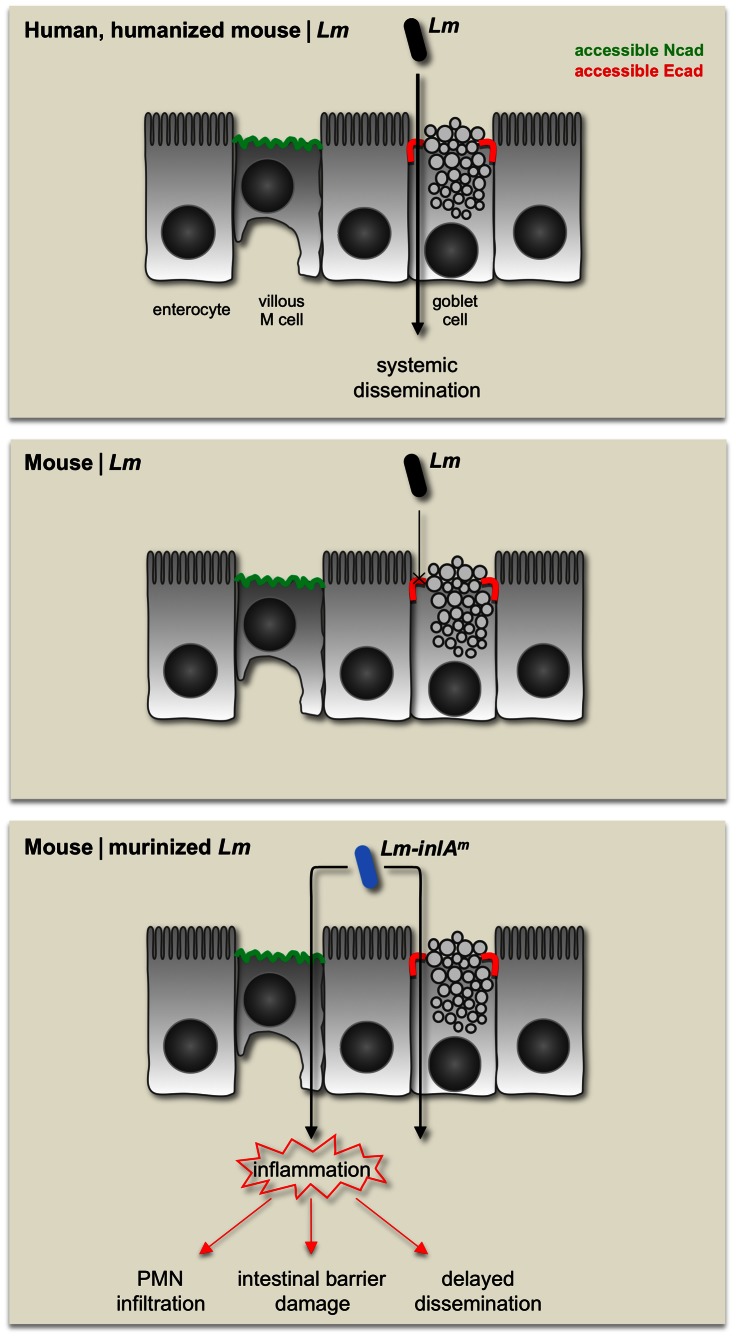
Revisited model of InlA^m^-expressing bacteria at the intestinal epithelium. In humanized mEcad-expressing E16P mouse, as well as in human, *Lm* targets accessible Ecad around intestinal goblet cells to cross intestinal epithelium, without inducing significant intestinal response. The wt InlA of *Lm* does not interact with mEcad, thus limits the ability of *Lm* to cross wt mouse intestinal epithelium. Murinization of InlA enables *Lm*-*inlA^m^* to interact with mEcad and also Ncad of mouse. *Lm-inlA^m^* therefore invades not only the goblet cells but also the villous M cells expressing accessible Ncad in the intestinal epithelia of mice. Targeting of villous M cells, which is not observed in *Lm*-infected E16P KI mice and is not predicted to occur in humans, results in severe intestinal inflammation which induces polymorphonuclear neutrophil (PMN) infiltration, intestinal barrier damage and a delayed systemic dissemination in wt mice, all of which are not observed in human listeriosis and *Lm*-infected humanized mice.

Importantly, we also uncover that InlA^m^ is able to recruit Ncad and mediate Ncad-dependent internalization. This artifactual interaction translates *in vivo* into InlA^m^-dependent targeting of villous M cells, intestinal inflammatory responses, disruption of intestinal barrier integrity and delayed bacterial systemic dissemination in wt mice, as well as in humanized mice. Such stricking phenotypes are not observed in humanized mice orally-inoculated with wt *Lm*, suggesting that they depend on InlA^m^-Ncad interaction and invasion of villous M cells, but not on InlA^m^-Ecad interaction and invasion of goblet cells (Figure 8). It is important to note that these phenotypes are also present in E16P KI and hEcad Tg mice infected with *Lm-inlA^m^*, indicating that intestinal inflammation is a direct consequence of InlA^m^-mediated intestinal invasion, and proving that the absence of inflammation in *Lm*-infected humanized mice is not a side effect of mouse humanization, but is a genuine property of InlA-dependent intestinal invasion. These results are in agreement with the observation by Wollert *et al.* that infection with *Lm*-*inlA^m^* leads to severe intestinal inflammation and tissue damage in wt mice [16], and with our earlier observation that InlA has little impact on *Lm* intestinal responses in mice permissive to InlA-Ecad interaction [6], [27]. This indicates that the murinization of InlA, in addition to broadening the host range of *Lm*, also extends its receptor repertoire to another member of the classical cadherin family, Ncad, therefore modifying its cell tropism, host responses and the dynamics of infection.

The engineering of InlA^m^ was based on the rational protein design of a modified InlA that would increase InlA-hEcad binding affinity [16]. Indeed, S192N and Y369S substitutions in InlA lead to a 6,700-fold increase in the binding affinity of InlA to hEcad [16]. Here we have shown that this does not translate into increased invasion of hEcad-expressing cells. Before drawing this conclusion, we ensured that the BHK21 cell line we used does not express other cadherins than the one we intended to study. A possible reason for the observed increased level of invasion of *Lm-inlA^m^* in Caco-2 cells observed by Wollert *et al.* is the coexpression of Ecad and Ncad in these cells [21]. These results suggest that InlA-hEcad interaction, although it is of relatively low affinity (K_D_ = 8±4 µM) [16], has been naturally selected to mediate an optimal level of infection.

We have shown that InlB, another major invasion protein of *Lm*, does not play a significant role for the crossing of the intestinal barrier [23]. In contrast, InlB has been reported to promote *Lm* expressing InlA^m^ to invade intestinal villi [34]. Our results shed light onto these apparent contradictory results and raise the possibilty that InlA^m^-Ncad mediated invasion of villous M cells may involve the InlB pathway.

*Shigella flexneri*, the etiological agent of bacillary dysentery is associated with strong polymorphonuclear infiltration, severe local inflammation, disruption of intestinal barrier integrity, yet no systemic dissemination [35], [36]. In contrast, listeriosis in human and humanized mice is characterized by the paucity of intestinal symptoms, the absence of polymorphonuclear intestinal infiltration, little local inflammation, the absence of intestinal barrier disruption, but systemic dissemination [6], [27], [36], [37]. We have demonstrated that *Lm*-*inlA^m^* triggers pro-inflammatory response and disrupts epithelial integrity in intestinal tissue of wt and humanized mice, and exhibits a delayed systemic dissemination, compared to *Lm*-infected humanized mice. These observations strongly suggest that the targeting of villous M cells by InlA^m^-expressing bacteria triggers pro-inflammatory host responses which contain bacterial invasion but lead to intestinal epithelium damages. This fits with the observation that antigen delivery via villous M cells stimulates immune reponses [38]. Like InlA^m^, Als3 is a *Candida albicans* invasin that binds both Ecad and Ncad to invade host cells [39]. *Candida albicans* has been shown to favor gut inflammation and promotes food allergy accompanied by gut epithelial barrier hyperpermeability, the underlying mechanisms of which are so far unclear [40], [41]. Our study indicates that *Candida albicans* may use Als3 to target Ncad-positive villous M cells, and thereby trigger intestinal inflammation. The specific functions of villous M cells remain poorly understood, yet villous M cells are a particularly abundant constituent of the intestinal epithelium. Our results show that InlA^m^- and Als3-expressing microorganisms would be particularly instrumental to study villous M cell functions.

Repeated infection of mice *in vivo* or mouse cells *in vitro* allows the obtention of “murinized” pathogens adapted to the mouse. Despite the great adaptability of microbes, evolutionary constraints limit pathogen variability [42]. A mutation beneficial under certain environmental conditions may end up as disadvantageous in another, highlighting the fine-tuning of host-microbe interactions. The structure-based rational design of InlA^m^ was proposed as a subtle and elegant way to electively “murinize” a microbial ligand with least impact on the pathogen. However, we provide here evidence that the rationally designed InlA^m^ has gained the unfortunate ability to interact with another surface protein than its cognate receptor Ecad. Even though InlA^m^ mediates *Lm* crossing of the intestinal barrier, a phenotype which is strictly dependent on InlA-Ecad interaction, the way by which *Lm* crosses the intestinal barrier in an InlA^m^-dependent manner differs from what observed with wt *Lm* in humanized mice and humans, as does the resulting infection process. This illustrates that murinization of human-specific pathogens, although an elegant and rational approach, may unfortunately mislead rather than ease the understanding of human infectious diseases' pathophysiology. Caution must therefore be exercised before engineering and using “murinized” pathogens to study human infectious diseases.

## Materials and Methods

### Bacterial and cell culture

Bacterial strains, plasmids and primers are listed in Table S1. Note that the sequences of *inlA*, *inlA^m^* in *Lm* and in *Li* were confirmed by sequencing, as well as the integration sites of *inlA* and *inlA^m^* in *Li* and the deletion site of *inlA* in *Lm*. *Listeria* and *Escherichia coli* strains were respectively cultivated in BHI and LB at 37°C with shaking at 180 rpm. To deliver plasmids into *Li*, *E. coli* S17-1 (colistin and nalidixic acid sensitive) cells were transformed with the plasmids followed by conjugation with *Li* (colistin and nalidixic acid resistant). Mammalian cell lines used in this study were routinely cultured at 37°C in 5% CO_2_. Except for the culture medium for BHK21 which was supplemented with 5% fetal bovine serum, all the cell culture media were supplemented with 10% fetal bovine serum. Human epithelium LoVo cells were cultured in F12K nutrient GlutaMax medium. Mouse epithelium Nme cells were cultured in DMEM GlutaMax medium supplemented with 10 µg/ml insulin. Mouse CT26 and guinea pig 104C1 cells were cultured in RPMI1640 GlutaMax medium supplemented with HEPES buffer and sodium pyruvate. Human HeLa cells were cultured in MEM GlutaMax medium. Hamster BHK21 cells were cultured in GMEM GlutaMax medium supplemented with tryptose phosphate buffer and HEPES buffer. All the culture medium and related chemicals were purchased from Gibco (Invitrogen). Transient transfection of mammalian cells was performed with jetPRIME transfection kit (Polyplus transfection). The scrambled (sc-37007) and mouse Ncad specific siRNAs (sc-35999) were purchased from Santa Cruz. For the transfection of siRNAs, mouse CT26 cells were seeded into the 24-well plates for 1 day and then transfected with scrambled siRNAs (25 nM) or mNcad-specific siRNAs (25 nM) followed by 1 day incubation and replacement of transfection medium with growth medium another 1 day of incubation before infection. For the transfection of plasmid DNAs, BHK21 cells were transiently transfected with pcDNA3 expression vector harboring the cDNAs of each cadherin (1 µg DNA for each well in a 24-well plate) followed by 2 days incubation before infection.

### Construction of plasmids

The strategy to express *inlA* or *inlA^m^* in *Li* is as described based on integrative plasmid pAD containing a constitutive promoter [43]. The primers EagI_UTRhly-F and UTRhly-R were used to amplify the *hly* 5′ UTR of *Lm* EGDe. Full length of *inlA* and *inlA^m^* were amplified from the genomic DNA of *Lm* EGDe and *Lm*-*inlA^m^*, respectively, with the primers UTRhly_inlA-F and SalI_inlA-R2. The resulting PCR products were ligated to *hly* 5′ UTR by splicing-by-overlap-extension (SOE) PCR. The final SOE PCR products, containing the entire *hly* 5′ UTR sequence fused to the start codon of the *inlA* (*hly* 5′ UTR-*inlA*) or *inlA^m^*, (*hly* 5′ UTR-*inlA^m^*), were then cloned in pCR-Blunt (Invitrogen) and verified by sequencing. Plasmids containing correct sequence and pAD-cGFP were digested by EagI and SalI. The backbone of pAD-cGFP was ligated with *hly* 5′ UTR-*inlA* and *hly* 5′ UTR-*inlA^m^* to form pAD-*inlA* and pAD-*inlA^m^*.

The mouse N-cadherin (mNcad) cDNA was bought from Open Biosystems (Thermo Scientific) and the cDNAs of mouse P-cadherin (mPcad) and mouse VE-cadherin (mVEcad) were from Riken Fantom Clones (Dnaform). To form pcDNA3-mNcad, mNcad cDNA was cloned into EcoRI-NotI site of pcDNA3. The plasmids pcDNA3-mPcad and pcDNA3-mVEcad were constructed by inserting mPcad and mVEcad cDNAs into NotI-KpnI site of pcDNA3, respectively.

### Invasion assay

Cell suspensions from confluent monolayers were seeded at a concentration of 5×10^4^ cells per well in 24-well tissue culture plates and grown for 40–48 hr in an antibiotics-free medium at 37°C. *Lm* and *Li* strains were grown to OD600 at 0.8 and 0.6 in BHI, respectively. Bacterial culture were then washed with PBS and diluted in cell culture medium without serum. Bacterial suspensions were added to the cells at a multiplicity of infection (MOI) of approximately 50 and incubated for 1 hr. Following wash with complete medium, 10 µg/ml of gentamicin was added to kill the extracellular bacteria for 1 hr. The cells were then washed by complete medium and PBS, and homogenized in PBS supplemented with 0.4% Triton X-100, followed by serial dilution and colony forming units (CFUs) counting. For cadherin recruitment assay, the procedure was the same as the invasion assay except that the cell attachment buffer (HEPES 20 mM, NaCl 150 mM, glucose 50 mM, MgCl_2_ 1 mM, CaCl_2_ 2 mM, MnCl_2_ 1 mM, 0.1% BSA) was used for infection and PBS (Ca^2+^/Mg^2+^) (Gibco) was applied to wash the non-attached bacteria stringently followed by fixation.

### Animals

Eight to 10-week old C57BL/6 female mice (JANVIER) and isogenic mEcad E16P KI female mice were food restricted overnight but allowed free access to water. *Lm* culture was prepared as described [6], and inoculated with a feeding needle intragastrically [44]. Mice were then immediately allowed free access to food and water. All the procedures were in agreement with the guidelines of the European Commission for the handling of laboratory animals, directive 86/609/EEC (http://ec.europa.eu/environment/chemicals/lab_animals/home_en.htm) and were approved by the Animal Care and Use Committee of the Institut Pasteur, as well as by the ethical committee of “Paris Centre et Sud” under the number 2010-0020.

### Immunofluorescence labeling and immunoblotting

Preparation of tissue sections and whole mount tissues were as described [9], [25]. The following antibodies and fluorescent probes were used for immunostaining and Western blot: anti-hEcad clone HECD-1 mouse monoclonal antibody (Invitrogen), anti-mEcad clone ECCD-2 rat monoclonal antibody (Invitrogen), anti-β-actin clone AC-15 mouse monoclonal antibody (Sigma), anti-Ncad clone 32/N-cadherin mouse monoclonal antibody (BD), anti-Ncad clone GC-4 mouse monoclonal antibody (Sigma), anti-pan cadherin clone CH-19 monoclonal antibody (Sigma), anti-M cell clone NKM 16-2-4 rat monoclonal antibody (Miltenyl Biotec), R6 anti-*Li* rabbit polyclonal antibody and R11 anti-*Lm* rabbit polyclonal antibody [45], Rat anti-mouse Ly-6G (BD), wheat germ agglutinin (WGA) conjugated with Alexa Fluor 647 (Jackson ImmunoResearch), Alexa Fuor 488 goat anti-rabbit (Invitrogen), Alexa Fluor 488 or Alexa Fluor 546 goat anti-mouse (Invitrogen), Alexa Fluor 647 donkey anti-rat (Jackson ImmunoResearch), Alexa Fluor 546 goat anti-rat (Invitrogen), Cy3-conjugated streptavidin (Jackson ImmunoResearch) and Hoechst 33342 (Invitrogen).

### Biotin penetration experiment

Biotin was used as a molecule to address the integrity of intestinal epithelium as described previously [33]. Briefly, 2 mg/ml of EZ-link Sulfo-NHS-Biotin (Pierce) in PBS was slowly injected into the lumen of ileum loop via the open end adjacent to cecum immediatedly after removal of the entire ileum. After 3 min, the loop was opened followed by PBS wash and 4% paraformaldehye fixation.

### Intestinal tissue genes expression quantification

Four mice for each condition were sacrificed 2 days post infection. 1 cm-long of ileal loop of each animal was applied for RNA extraction. The RNA isolation, reverse transcription and quantitative real time PCR (qRT-PCR) were performed as described [46]. Primers used for qRT-PCR were pre-designed, validated RT^2^ qPCR primer pairs (SABioSciences, Qiagen) as follows: IFNG (IFN-γ, PPM03121A), IL1B (IL-1β, PPM03109F) and GAPDH (PPM02946E).

### Statistical analysis

Values are expressed as mean + SD. Statistical comparisons were made using the unpaired Student's *t* test, Mann-Whitney *u* test or the χ^2^ test as indicated. *p* values<0.05 were considered significant. Significant differences are marked with an asterisk for *p*<0.05, two asterisks for *p*<0.01, three asterisks for *p*<0.001 and four asterisks for *p*<0.0001.

## Supporting Information

Figure S1 **Murinization of InlA allows bacteria to recruit cadherin in Ecad-negative mouse cells, related to** **Figure 2** **.** (A) Amino acids sequence alignment of first extracellular domains (EC1) of type I classical cadherins and mouse VE-cadherin, a type II classical cadherin. (B) Recruitment of mNcad in CT26 cells was performed as described in Figure 1. The coverslips were stained with the anti-*Li* antibody and anti-Pan-cadherin (Pan-cad) antibody. Right panels show the boxed regions of separated channels and merge, demonstrating the recruitment of cadherin proteins specifically by *Li-inlA^m^*. Scale bar, 20 µm.
(PDF)

Figure S2 **Murinization of InlA promotes bacterial entry into Ecad-negative, Ncad-positive human and guinea pig cells, related to** **Figure 2** **.** Human HeLa cells and guinea pig 104C1 cells are Ecad-negative and Ncad-positive cells. Cell invasion ability was evaluated by counting intracellular gentamicin resistant bacteria following 1 hr of infection (MOI 50) and 1 hr of gentamicin (10 µg/ml) incubation. No difference in bacterial entry is seen between *Lm* and its isogenic *inlA* null mutant (*Lm*Δ*inlA*), whereas the *Lm* harboring *inlA^m^* of which chromosomal *inlA* is replaced by *inlA^m^* (*Lm*-*inlA^m^*) promoted bacterial entry into both HeLa and 104C1 cells. Values are expressed as a mean + SD (n = 3). Statistical analysis was performed with the unpaired Student's *t* test.
(PDF)

Figure S3 **Ncad expression is detected on the apical pole of villous M cells in E16P KI mice, related to** **Figure 4** **.** Intestinal tissue of wt mice was fixed and stained for luminally accessible (acc) Ncad with antibody against extracellular domain of Ncad (clone GC4) before tissue permeabilization, M cells with NKM 16-2-4 antibody, WGA and nuclei after tissue permeabilization. Projection of a 30 µm thick reconstructed intestinal villus (A) and one xy plane (B) are shown. Right panels show separated channels and merge of boxed regions in (B), showing Ncad on the apical side of NKM 16-2-4-positive cells. See also S7. NKM 16-2-4 antibody is a monoclonal antibody raised against α(1,2) fucose moiety in absence of neighboring sialic acids, a specific marker on M cells surface. WGA was used to stain the mucus of goblet cells and cell membrane. Scale bar, 20 µm.
(PDF)

Figure S4 ***Lm-inlA^m^*** **target goblet cells and villous M cells in wt mice, related to** **Figure 5** **.** The intestinal ileum was taken from wt or E16P KI mice orally inoculated by 10^10^ *Lm* or *Lm-inlA^m^* at 5 hr post infection. The intestinal tissues were fixed. Vibratome sections were stained with WGA for goblet cells, NKM 16-2-4 monoclonal antibody for M cells, and for bacteria and nuclei. (A and B) The confocal Z-plane of an ileal villus from *Lm-inlA^m^* infected wt mice demonstrates that *Lm-inlA^m^* was able to target goblet cells (A, see also Figure S5A and Movie S5) and villous M cells (B, see also Figure S5B, and Movie S6). Right panels show separated channels and merge of boxed regions, showing the location of bacteria in villous epithelia. (C) The confocal Z-plane of an ileal villus from *Lm* infected E16P KI mouse shows that *Lm* targeted goblet cells (see also Figure S5C and Movie S7). (D) Relative location of bacteria in mice intestinal epithelia of villi is shown. The total number of *Lm-inlA^m^* in wt mice intestinal villi epithelia was set to 100. 20 villi from two mice ileal loops were counted in each set. Scale bar, 20 µm.
(PDF)

Figure S5 **Intracellular location of bacteria targeting goblet and villous M cells, related to** **Figure 5** **.** Orthogonal views of the infected cells in wt mice infected with *Lm-inlA^m^* (A and B, related to Figures S4A and B, respectively) and in E16P KI mice infected by *Lm* (C, related to Figure S4C) presented in Figure S5 were shown. These images demonstrate that the bacteria highlighted in the Figure S4 were intracelullar. See also Movies S5, S6 and S7.
(PDF)

Figure S6 ***Lm-inlA^m^*** **attached to villous M cells and** ***Lm-inlA^m^*** **having reached the lamina propria underlying villous M cells, related to** **Figure 5** **.** The intestinal ileum was taken from the wt mice orally inoculated by 10^10^ *Lm* or *Lm-inlA^m^* at 5 hr post infection. The intestinal tissues were fixed. Vibratome sections were stained with WGA for goblet cells, NKM 16-2-4 monoclonal antibody for M cells, and for bacteria and nuclei. Results shown are two different confocal Z-planes of an ileal villus from *Lm-inlA^m^* infected wt mice. *Lm-inlA^m^* was found to attach to the apical pole of villous M cell in the upper panel and reach the lamina propria in the lower panel. Scale bar, 20 µm.
(PDF)

Figure S7 **Respective invasive potential of** ***Lm*** **and** ***Lm*** **-** ***inlA*** **^m^ in orally inoculated E16P KI mice, related to** **Figure 6** **.** Mice were orally inoculated by 10^10^ bacteria for 2 (n = 6, upper panel) or 4 (n = 6, lower panel) days. Bacterial loads in the ileum loops of small intestine, the spleens and livers were shown. Statistical analysis was performed with the Mann-Whitney *u* test.
(PDF)

Figure S8 ***Lm*** **-** ***inlA^m^*** **induced neutrophil infiltration and compromised intestinal epithelial barrier integrity in E16P KI and hEcad Tg mice, related to** **Figure 7** **.** The intestinal ileum was taken from E16P KI and hEcad Tg mice orally inoculated by 10^10^ *Lm*Δ*inlA*, *Lm* and *Lm-inlA^m^* 24 hr (A to C) or 48 hr (D) post infection. (A) Anti-Ly6G antibody staining indicates neutrophils (red, highlighted by the open arrowheads). Tissues were stained for *Lm* (green, highlighted by the arrows) and counterstained with WGA (grey) for goblet cells and epithelia. Scale bar, 20 µm. (B) No obvious difference on neutrophil numbers was observed between *Lm*Δ*inlA* and *Lm* infection in hEcad Tg mice, whereas *Lm-inlA^m^* infection induced neutrophil infiltration in the intestinal villi compared to *Lm* in both E16P KI and hEcad Tg mice. (C) The number of bacteria in each infected villus was also quantified. Bacteria load of *Lm* in the intestinal villi was higher than that of *Lm-inlA^m^* in both E16P KI and hEcad Tg mice upon oral infection 24 hpi. In order to compare the result of *Lm-inlA^m^* with *Lm* in E16P KI mice, the data of *Lm*-infected E16P KI mice shown here in B and C were from figure 7B and C, respectively. Statistical analysis was done with Mann-Whitney *u* test (n = 20 villi from 2 mice). (D) Biotin was injected into ileum loop followed by PBS wash and fixation. Tissues were stained for *Lm* (green, highlighted by the arrows) and counterstained with WGA (grey) for goblet cells and epithelia. Biotin is located within lamina propria of the villi from *Lm*-*inlA^m^* infected mice but not *Lm* infected mice. Scale bar, 20 µm.
(PDF)

Movie S1 **Luminally accessible Ncad is expressed on the apical poles of villous M cells in wt mice, related to** **Figure 4** **.** Whole mount intestinal tissue of a wt mouse was stained before permeabilization for accessible mNcad (green) and NKM 16-2-4 for M cells (red), and after permeabilization for nuclei (blue) and WGA for goblet cells (grey). Intestinal villus is oriented with the villus tip facing the viewer. The luminally accessible apical surface of villous M cells is labeled with the anti-Ncad antibody. Images were acquired as a z stack by confocal microscopy and assembled as a three-dimensional reconstruction with Imaris software.
(MOV)

Movie S2 **Peyer's patch M cells do not express luminally accessible Ncad in wt mice, related to** **Figure 4** **.** Whole mount intestinal tissue of a wt mouse was stained before permeabilization for accessible mNcad (green) and NKM 16-2-4 for M cells (red), and after permeabilization for nuclei (blue) and WGA for goblet cells (grey). The luminally accessible apical surface of Peyer's patch M cells is not labeled with the anti-Ncad antibody. Intestinal Peyer's patch is oriented with the tip facing the viewer. Images were acquired as a z stack by confocal microscopy and assembled as a three-dimensional reconstruction with Imaris software.
(MOV)

Movie S3 **Luminally accessible Ncad is expressed on the apical poles of villous M cells in E16P KI mice, related to** **Figure 4** **.** Whole mount intestinal tissue of an E16P KI mouse was stained before permeabilization for accessible mNcad (green) and NKM 16-2-4 for M cells (red), and after permeabilization for nuclei (blue) and WGA for goblet cells (grey). Intestinal villus is oriented with the villus tip facing the viewer. The luminally accessible apical surface of villous M cells is labeled with the anti-Ncad antibody. Images were acquired as a z stack by confocal microscopy and assembled as a three-dimensional reconstruction with Imaris software.
(MOV)

Movie S4 ***Li-inlA^m^*** **targets both villous M cells and goblet cells in the intestinal villi upon oral inoculation of wt mice, related to** **Figure 5** **.** Ileal loop of a wt mouse orally infected by *Li-inlA^m^* was taken 5 hr post infection, followed by fixation and staining for *Li* (green), M cells (red), goblet cells (grey) and nuclei (blue) after permeabilization. Images were acquired and assembled as described for Movie S1.
(MOV)

Movie S5 ***Lm-inlA^m^*** **targets goblet cells in the intestinal villi upon oral inoculation of wt mice, related to** **Figure 5** **.** Ileal loop of a wt mouse orally infected by *Lm-inlA^m^* was taken 5 hr post infection, followed by fixation. Vibratome section was stained for *Lm-inlA^m^* (green), M cells (red), goblet cells (grey) and nuclei (blue) after permeabilization. Images were acquired and assembled as described for Movie S1.
(MOV)

Movie S6 ***Lm-inlA^m^*** **targets villous M cells in the intestinal villi upon oral inoculation of wt mice, related to** **Figure 5** **.** Ileal loop of a wt mouse orally infected by *Lm-inlA^m^* was taken 5 hr post infection, followed by fixation. Vibratome section was stained for *Lm-inlA^m^* (green), M cells (red), goblet cells (grey) and nuclei (blue) after permeabilization. Images were acquired and assembled as described for Movie S1.
(MOV)

Movie S7 ***Lm*** **targets goblet cells in the intestinal villi upon oral inoculation of E16P KI mice, related to** **Figure 5** **.** Ileal loop of a wt mouse orally infected by *Lm* was taken 5 hr post infection, followed by fixation. Vibratome section was stained for *Lm* (green), M cells (red), goblet cells (grey) and nuclei (blue) after permeabilization. Images were acquired and assembled as described for Movie S1.
(MOV)

Table S1 **Bacterial strains, plasmids and primers used in this study.**
(DOC)
