# Supplementary figures and images for: Murinization of Internalin Extends Its Receptor Repertoire, Altering Listeria monocytogenes Cell Tropism and Host Responses
Source: PLoS Pathog. 2013 May 30;9(5):e1003381. doi: 10.1371/journal.ppat.1003381 (PMC3667765; doi:10.1371/journal.ppat.1003381)

Figure S1

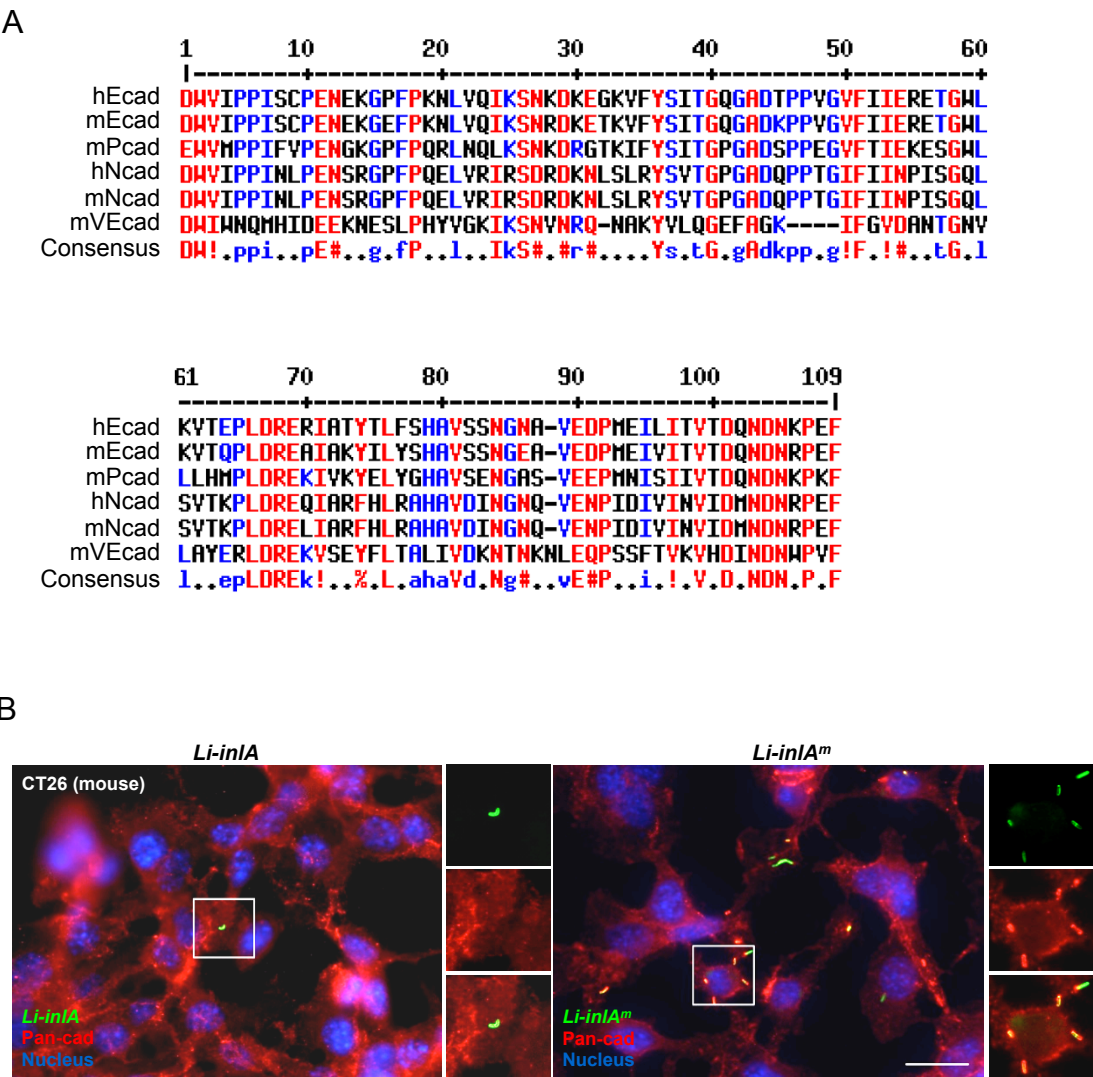

Supplement: Figure S1 — Murinization of InlA allows bacteria to recruit cadherin in Ecad-negative mouse cells, related to Figure 2 . (A) Amino acids sequence alignment of first extracellular domains (EC1) of type I classical cadherins and mouse VE-cadherin, a type II classical cadherin. (B) Recruitment of mNcad in CT26 cells was performed as described in Figure 1. The coverslips were stained with the anti-Li antibody and anti-Pan-cadherin (Pan-cad) antibody. Right panels show the boxed regions of separated channels and merge, demonstrating the recruitment of cadherin proteins specifically by Li-inlAm. Scale bar, 20 µm. (PDF) [file ppat.1003381.s001.pdf]

Figure S2

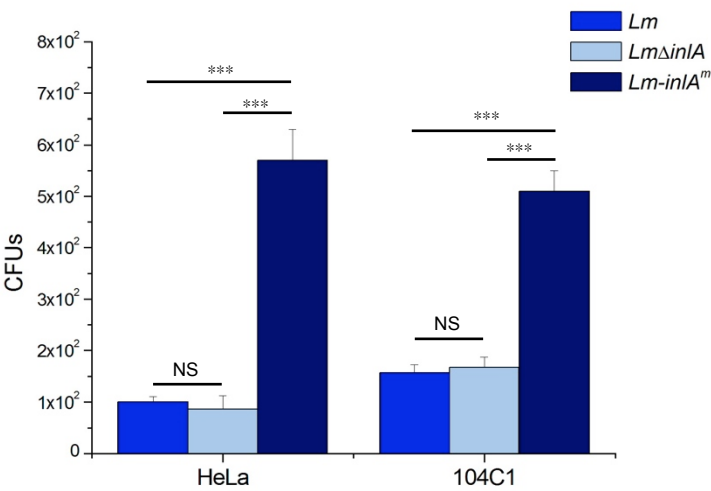

Supplement: Figure S2 — Murinization of InlA promotes bacterial entry into Ecad-negative, Ncad-positive human and guinea pig cells, related to Figure 2 . Human HeLa cells and guinea pig 104C1 cells are Ecad-negative and Ncad-positive cells. Cell invasion ability was evaluated by counting intracellular gentamicin resistant bacteria following 1 hr of infection (MOI 50) and 1 hr of gentamicin (10 µg/ml) incubation. No difference in bacterial entry is seen between Lm and its isogenic inlA null mutant (LmΔinlA), whereas the Lm harboring inlAm of which chromosomal inlA is replaced by inlAm (Lm-inlAm) promoted bacterial entry into both HeLa and 104C1 cells. Values are expressed as a mean + SD (n = 3). Statistical analysis was performed with the unpaired Student's t test. (PDF) [file ppat.1003381.s002.pdf]

Figure S3

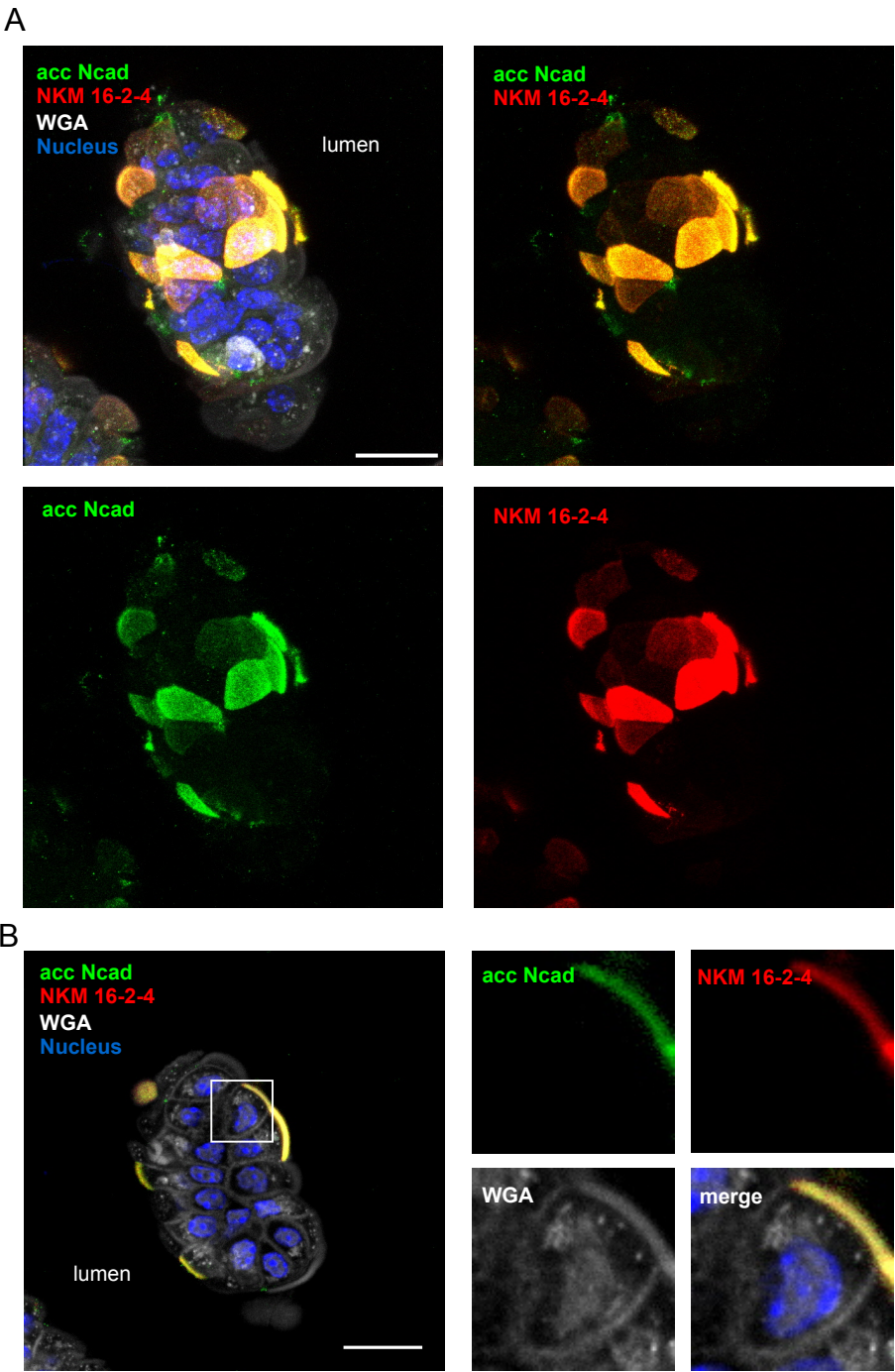

Supplement: Figure S3 — Ncad expression is detected on the apical pole of villous M cells in E16P KI mice, related to Figure 4 . Intestinal tissue of wt mice was fixed and stained for luminally accessible (acc) Ncad with antibody against extracellular domain of Ncad (clone GC4) before tissue permeabilization, M cells with NKM 16-2-4 antibody, WGA and nuclei after tissue permeabilization. Projection of a 30 µm thick reconstructed intestinal villus (A) and one xy plane (B) are shown. Right panels show separated channels and merge of boxed regions in (B), showing Ncad on the apical side of NKM 16-2-4-positive cells. See also S7. NKM 16-2-4 antibody is a monoclonal antibody raised against α(1,2) fucose moiety in absence of neighboring sialic acids, a specific marker on M cells surface. WGA was used to stain the mucus of goblet cells and cell membrane. Scale bar, 20 µm. (PDF) [file ppat.1003381.s003.pdf]

Figure S4

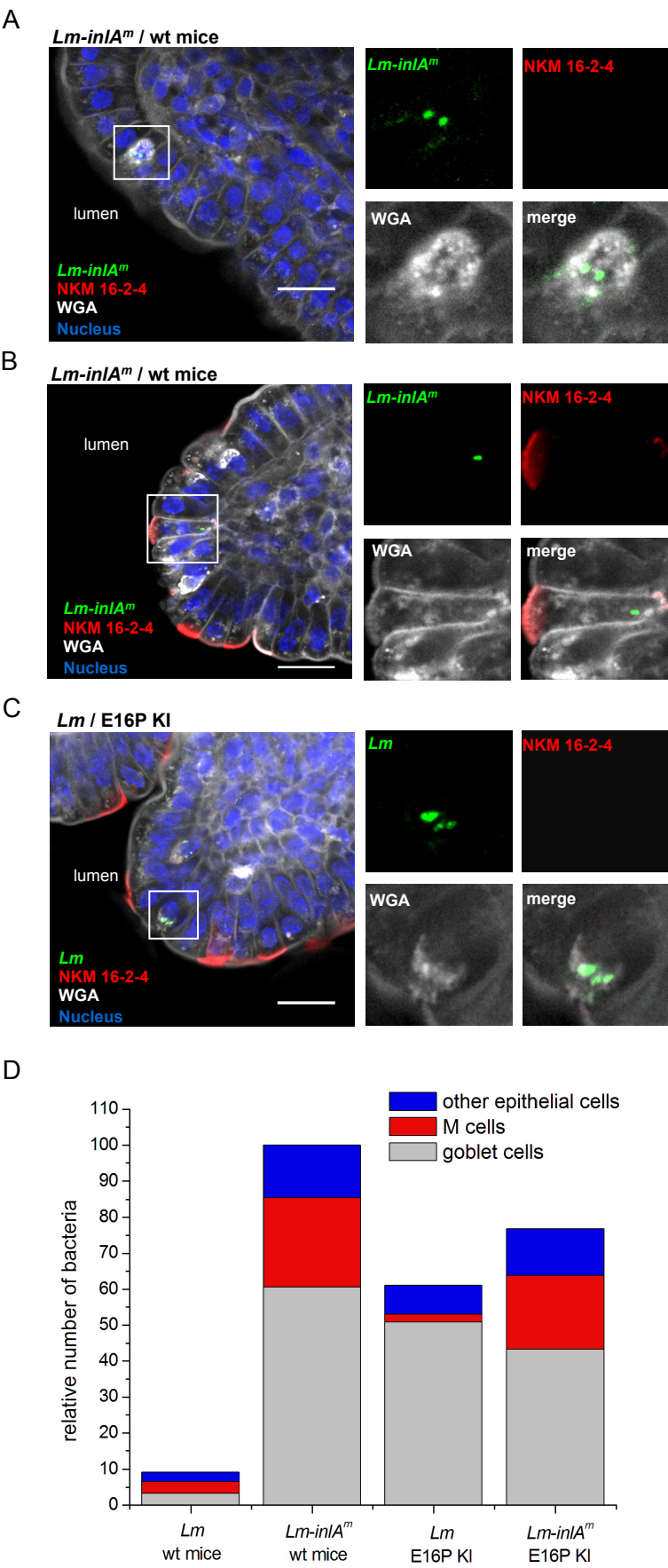

Supplement: Figure S4 — Lm-inlAm target goblet cells and villous M cells in wt mice, related to Figure 5 . The intestinal ileum was taken from wt or E16P KI mice orally inoculated by 1010 Lm or Lm-inlAm at 5 hr post infection. The intestinal tissues were fixed. Vibratome sections were stained with WGA for goblet cells, NKM 16-2-4 monoclonal antibody for M cells, and for bacteria and nuclei. (A and B) The confocal Z-plane of an ileal villus from Lm-inlAm infected wt mice demonstrates that Lm-inlAm was able to target goblet cells (A, see also Figure S5A and Movie S5) and villous M cells (B, see also Figure S5B, and Movie S6). Right panels show separated channels and merge of boxed regions, showing the location of bacteria in villous epithelia. (C) The confocal Z-plane of an ileal villus from Lm infected E16P KI mouse shows that Lm targeted goblet cells (see also Figure S5C and Movie S7). (D) Relative location of bacteria in mice intestinal epithelia of villi is shown. The total number of Lm-inlAm in wt mice intestinal villi epithelia was set to 100. 20 villi from two mice ileal loops were counted in each set. Scale bar, 20 µm. (PDF) [file ppat.1003381.s004.pdf]

Figure S5

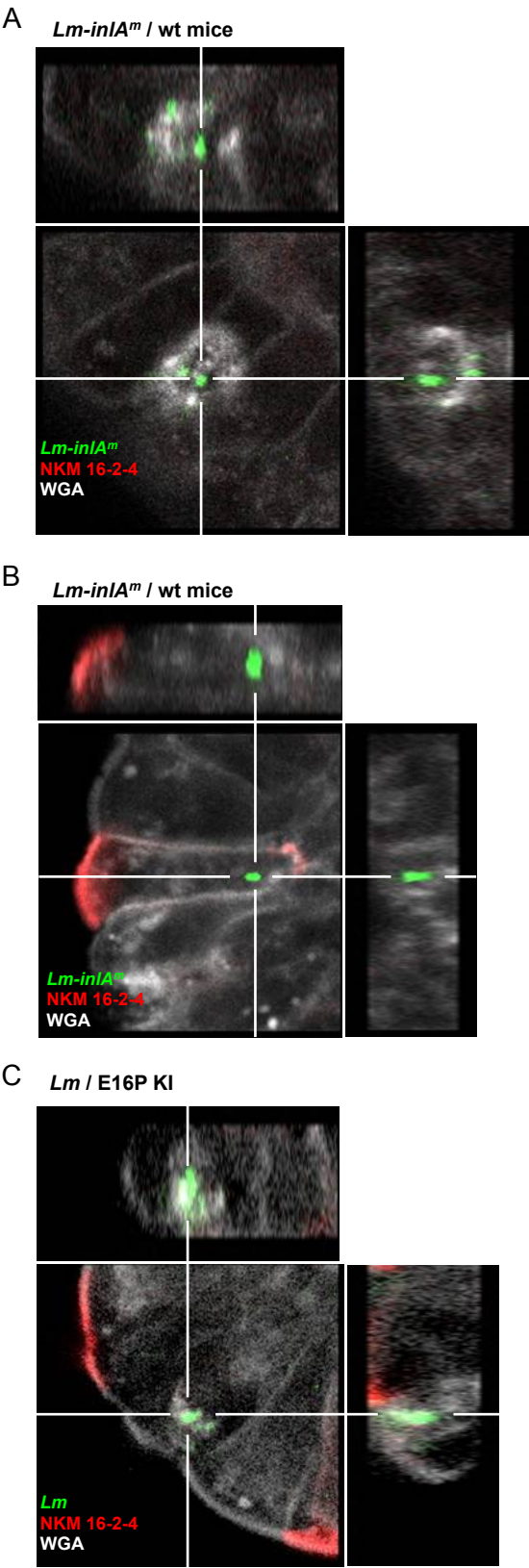

Supplement: Figure S5 — Intracellular location of bacteria targeting goblet and villous M cells, related to Figure 5 . Orthogonal views of the infected cells in wt mice infected with Lm-inlAm (A and B, related to Figures S4A and B, respectively) and in E16P KI mice infected by Lm (C, related to Figure S4C) presented in Figure S5 were shown. These images demonstrate that the bacteria highlighted in the Figure S4 were intracelullar. See also Movies S5, S6 and S7. (PDF) [file ppat.1003381.s005.pdf]

Figure S6

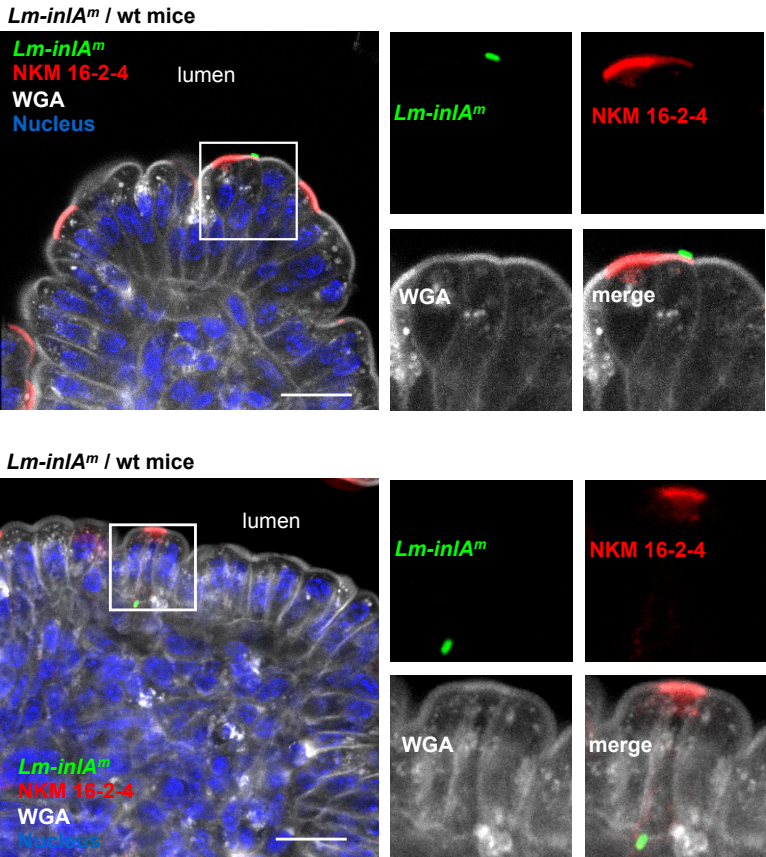

Supplement: Figure S6 — Lm-inlAm attached to villous M cells and Lm-inlAm having reached the lamina propria underlying villous M cells, related to Figure 5 . The intestinal ileum was taken from the wt mice orally inoculated by 1010 Lm or Lm-inlAm at 5 hr post infection. The intestinal tissues were fixed. Vibratome sections were stained with WGA for goblet cells, NKM 16-2-4 monoclonal antibody for M cells, and for bacteria and nuclei. Results shown are two different confocal Z-planes of an ileal villus from Lm-inlAm infected wt mice. Lm-inlAm was found to attach to the apical pole of villous M cell in the upper panel and reach the lamina propria in the lower panel. Scale bar, 20 µm. (PDF) [file ppat.1003381.s006.pdf]

Figure S7

2 dpi

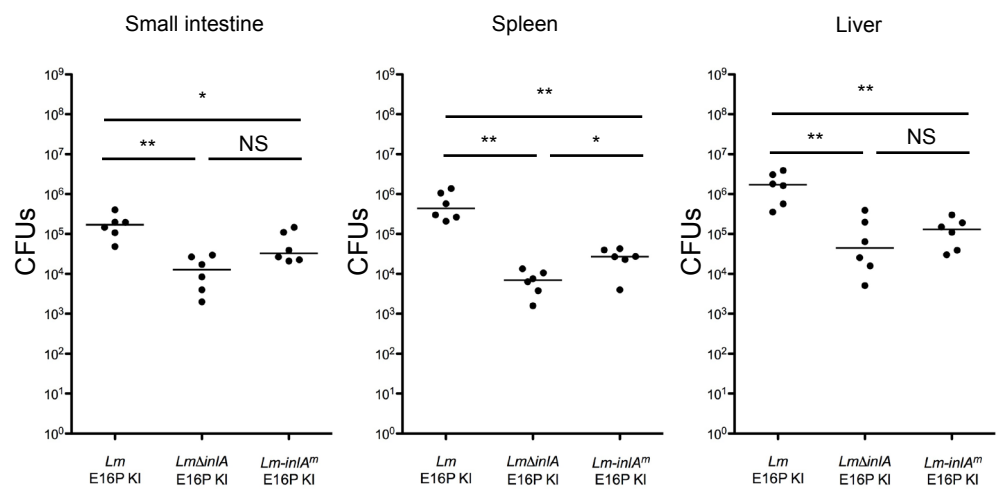

4 dpi

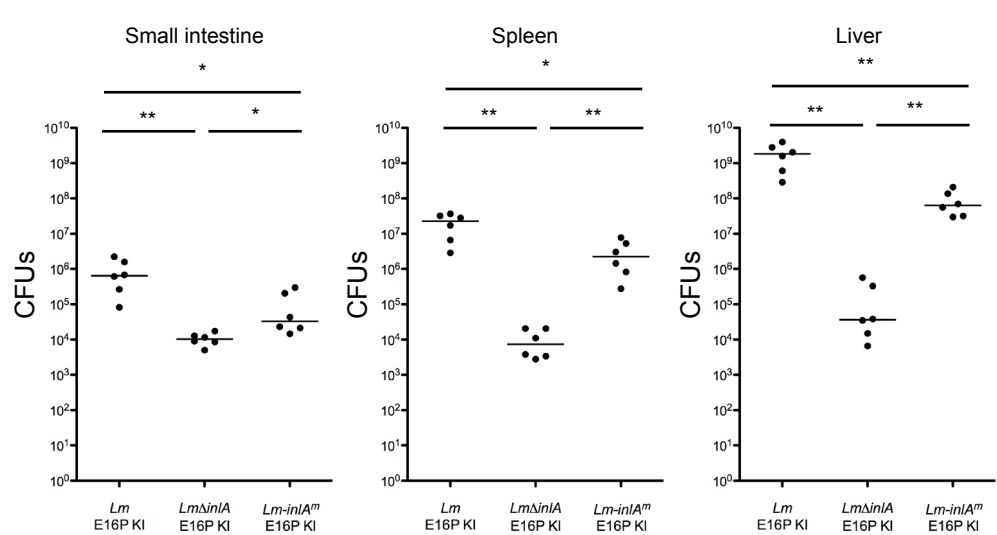

Supplement: Figure S7 — Respective invasive potential of Lm and Lm - inlA m in orally inoculated E16P KI mice, related to Figure 6 . Mice were orally inoculated by 1010 bacteria for 2 (n = 6, upper panel) or 4 (n = 6, lower panel) days. Bacterial loads in the ileum loops of small intestine, the spleens and livers were shown. Statistical analysis was performed with the Mann-Whitney u test. (PDF) [file ppat.1003381.s007.pdf]

Figure S8

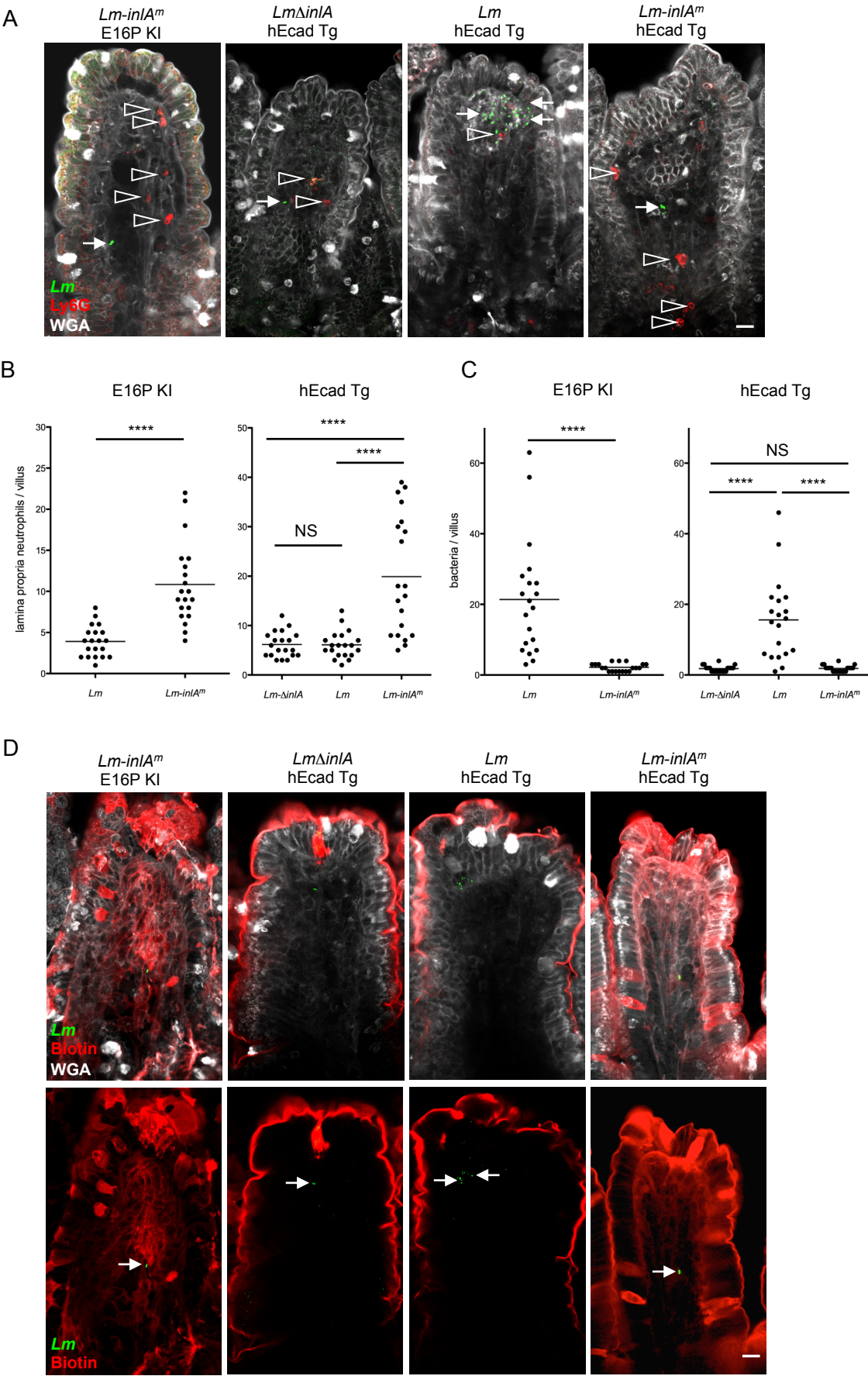

Supplement: Figure S8 — Lm - inlAm induced neutrophil infiltration and compromised intestinal epithelial barrier integrity in E16P KI and hEcad Tg mice, related to Figure 7 . The intestinal ileum was taken from E16P KI and hEcad Tg mice orally inoculated by 1010 LmΔinlA, Lm and Lm-inlAm 24 hr (A to C) or 48 hr (D) post infection. (A) Anti-Ly6G antibody staining indicates neutrophils (red, highlighted by the open arrowheads). Tissues were stained for Lm (green, highlighted by the arrows) and counterstained with WGA (grey) for goblet cells and epithelia. Scale bar, 20 µm. (B) No obvious difference on neutrophil numbers was observed between LmΔinlA and Lm infection in hEcad Tg mice, whereas Lm-inlAm infection induced neutrophil infiltration in the intestinal villi compared to Lm in both E16P KI and hEcad Tg mice. (C) The number of bacteria in each infected villus was also quantified. Bacteria load of Lm in the intestinal villi was higher than that of Lm-inlAm in both E16P KI and hEcad Tg mice upon oral infection 24 hpi. In order to compare the result of Lm-inlAm with Lm in E16P KI mice, the data of Lm-infected E16P KI mice shown here in B and C were from figure 7B and C, respectively. Statistical analysis was done with Mann-Whitney u test (n = 20 villi from 2 mice). (D) Biotin was injected into ileum loop followed by PBS wash and fixation. Tissues were stained for Lm (green, highlighted by the arrows) and counterstained with WGA (grey) for goblet cells and epithelia. Biotin is located within lamina propria of the villi from Lm-inlAm infected mice but not Lm infected mice. Scale bar, 20 µm. (PDF) [file ppat.1003381.s008.pdf]
