# Supplementary material for: Murinization of Internalin Extends Its Receptor Repertoire, Altering Listeria monocytogenes Cell Tropism and Host Responses
Source: PLoS Pathog. 2013 May 30;9(5):e1003381. doi: 10.1371/journal.ppat.1003381 (PMC3667765; doi:10.1371/journal.ppat.1003381)
Supplement: Table S1 — Bacterial strains, plasmids and primers used in this study. (DOC) [file ppat.1003381.s016.doc]

**Supporting Information**

Table S1. Bacterial strains, plasmids and primers used in this study

| Bacteria strains | Description | Source |
| --- | --- | --- |
| *Lm* | EGDe wild-type *Listeria monocytogenes* strain | [1]  ATCC-BAA-679 |
| *Lm*-*inlA^m^* | Isogenic EGDe with mutated residues S192N and Y369S by mutagenesis of endogenous *Lm* *inlA* gene | [1] |
| *Lm*Δ*inlA* | Isogenic EGDe *inlA* in-frame deletion mutant | [2] |
| *Li* | *Listeria innocua* | [3]  ATCC BAA-680 |
| *Li-egfp* | *Li* harboring pAD-cGFP in the chromosome | This study |
| *Li-inlA* | *Li* harboring pAD-*inlA* in the chromosome | This study |
| *Li-inlA^m^* | *Li* harboring pAD-*inlA^m^* in the chromosome | This study |
| Plasmids |  |  |
| pCR-Blunt | Blunt-end PCR cloning vector, Km^r^ | Invitrogen |
| pAD-cGFP | pPL2-P*hyper*-GFP, Cm^r^ | [4] |
| pAD-*inlA* | pPL2-P*hyper*-*inlA*, Cm^r^ | This study |
| pAD-*inlA^m^* | pPL2-P*hyper*-*inlA^m^*, Cm^r^ | This study |
| pcDNA3 | cDNA expression vector for mammalian cells, Amp^r^ | Invitrogen |
| pcDNA3-hEcad | pcDNA3-human Ecad cDNA | [5] |
| pcDNA3-mEcad | pcDNA3-mouse Ecad cDNA | [5] |
| pcDNA3-mNcad | pcDNA3-mouse Ncad cDNA | This study |
| pcDNA3-mPcad | pcDNA3-mouse Pcad cDNA | This study |
| pcDNA3-mVEcad | pcDNA3-mouse VEcad cDNA | This study |
| Primers | Sequence (5’ → 3’) |  |
| EagI_UTRhly-F | TCACGGCCGATAAAGCAAGCATATAATA |  |
| UTRhly-R | GGGTTTCACTCTCCTTCTACA |  |
| UTRhly_inlA-F | GGTTAAAAAATGTAGAAGGAGAGTGAAACCC  ATGAGAAAAAAACGATATGTATGGTT |  |
| SalI_inlA-R2 | CAGTCGACTTATTTACTAGCACGTGC |  |

Km^r^, kanamycin resistant (50 μg/ml) Amp^r^, ampicillin resistant (100 μg/ml); Cm^r^, chloramphenicol resistant (35 μg/ml for *E. coli* and 7 μg/ml for *Listeria*)

Restriction enzyme sites added to primers are underlined.

**Supporting Information References**

1. Monk IR, Casey PG, Hill C, Gahan CG (2010) Directed evolution and targeted mutagenesis to murinize *listeria monocytogenes* internalin A for enhanced infectivity in the murine oral infection model. BMC Microbiol 10: 318.

2. Lingnau A, Domann E, Hudel M, Bock M, Nichterlein T, et al. (1995) Expression of the *Listeria monocytogenes* EGD inlA and inlB genes, whose products mediate bacterial entry into tissue culture cell lines, by PrfA-dependent and -independent mechanisms. Infect Immun 63: 3896-3903.

3. Mengaud J, Ohayon H, Gounon P, Mège R-M, Cossart P (1996) E-Cadherin Is the Receptor for Internalin, a Surface Protein Required for Entry of *L. monocytogenes* into Epithelial Cells. Cell 84: 923-932.

4. Balestrino D, Hamon MA, Dortet L, Nahori MA, Pizarro-Cerda J, et al. (2010) Single-cell techniques using chromosomally tagged fluorescent bacteria to study *Listeria monocytogenes* infection processes. Appl Environ Microbiol 76: 3625-3636.

5. Lecuit M, Dramsi S, Gottardi C, Fedor-Chaiken M, Gumbiner B, et al. (1999) A single amino acid in E-cadherin responsible for host specificity towards the human pathogen Listeria monocytogenes. Embo J 18: 3956-3963.
